# Research on healthcare data sharing in the context of digital platforms considering the risks of data breaches

**DOI:** 10.3389/fpubh.2024.1438579

**Published:** 2024-11-06

**Authors:** Shizhen Bai, Jinjin Zheng, Wenya Wu, Dongrui Gao, Xiujin Gu

**Affiliations:** School of Management, Harbin University of Commerce, Harbin, China

**Keywords:** anonymous information sharing, medical data, data breaches, medical institution, information sharing, digital platform

## Abstract

**Background:**

Within China's healthcare landscape, the sharing of medical data has emerged as a pivotal force propelling advancements in the insurance sector and enhancing patient engagement with healthcare services. However, medical institutions often exhibit reluctance toward data sharing due to apprehensions regarding data security and privacy safeguards. To navigate this conundrum, our research introduces and empirically validates a model grounded in evolutionary game theory, offering a robust theoretical framework and actionable strategies for facilitating healthcare data sharing while harmonizing the dual imperatives of data utility and privacy preservation.

**Methods:**

In this paper, we construct an evolutionary game model involving medical institutions, big data innovation platforms, and insurance companies within the context of digital platforms. The model integrates exogenous causes of data breaches, endogenous causes of data breaches, compensation payments, government penalties, subsidies, unreasonable fees, claims efficiency, and insurance fraud.

**Results:**

The stability analysis of the evolutionary game identifies eight equilibrium points among medical institutions, platforms, and insurance companies. Numerical simulations demonstrate convergence toward strategy *E*_7_ = (0, 0, 1), suggesting a trend for medical institutions to adopt a fully anonymous information-sharing strategy, platforms to implement strict regulation, and insurance companies to opt for an auditing approach. Sensitivity analysis reveals that the parameters selected in this study significantly influence the players' behavioral choices and the game's equilibria.

**Conclusions:**

When breaches occur, medical institutions tend to seek co-sharing between platforms and insurance companies. This promotes enhanced regulation by platforms and incentivizes insurance companies to perform audits. If the responsibility for the breach is attributed to the platform or the insurance company, the liability sharing system will push healthcare organizations to choose a fully anonymous information sharing strategy. Otherwise, medical institutions will choose partially anonymous information sharing for more benefits. In case of widespread data leakage, the amount of compensation shall augment, and the role of compensation shall replace the role of government supervision. Then, the government shall penalize them, which shall reduce the motivation of each subject.

## 1 Introduction

In the contemporary digital era, the significance of healthcare data sharing has become increasingly prominent. This practice not only fosters advancements in medical research but also substantially enhances the quality and efficiency of healthcare services. As highlighted by the New England Journal of Medicine, effective utilization of shared data enables healthcare organizations to better comprehend disease trends and devise more precise prevention strategies, thereby reducing disease incidence. For instance, during the global response to Coronavirus Disease 2019 (COVID-19), international research collaborations expedited vaccine development through the sharing of viral genetic sequence information. In the United States, Blue Cross Blue Shield employs data analytics to identify high-risk patients and implement preventive interventions for chronic diseases, thereby improving patient quality of life and mitigating long-term healthcare expenditures. The landscape of health insurance data sharing, however, exhibits considerable variation across nations. The U.S. predominantly relies on a private insurance framework and safeguards personal health information via the Health Insurance Portability and Accountability Act (HIPAA), albeit with more restricted protections for data subjects' rights compared to the European Union. Conversely, the EU's General Data Protection Regulation (GDPR) enforces stringent standards concerning data transparency, data subject rights, and accountability for data processors, especially regarding cross-border data transfers. On January 17, 2024, the Shanghai Big Data Center, in collaboration with the Shanghai Medical Insurance Center and the China Insurance Science and Technology Federation, inaugurated the Shanghai Medical Insurance Big Data Innovation Laboratory. This initiative aims to foster the integration and sharing of medical and commercial insurance data, thereby enhancing the exploration and application of commercial health insurance in actuarial research, product development, and service innovation. Medical insurance data are derived from a subset of medical data, which is relatively more open compared to the more sensitive and complex medical data that involve patient privacy and may necessitate individual user consent (1). The implications of patient privacy breaches present substantial concerns for medical data custodians, notably healthcare institutions. Such incidents can result in stringent penalties for data custodians, fostering an environment of reticence regarding data sharing due to the looming threat of legal liabilities (2). Addressing the concerns of medical institutions regarding data leakage and enhancing their willingness to share information is the core issue of this paper.

The advent of anonymous information sharing has paved the way for the exchange of data among medical institutions. In China, the National Health Commission has championed the establishment of a universal health information platform that facilitates cross-regional and cross-organizational medical data sharing through anonymization. This initiative not only enhances the efficiency of healthcare services but also furnishes critical data support for epidemic prevention, control, and epidemiological investigations. When managing medical data, insurance companies must adhere to relevant laws and regulations while ensuring data security and protecting patient privacy. Furthermore, they should leverage information technology for effective risk management. Auditing, a crucial risk management tool, enables insurers to identify potential security threats and ensure compliance with industry standards and legal requirements. However, the cost and technological investment associated with auditing are significant considerations. Consequently, insurers must strike a balance between various factors to optimize business operations and risk management. Given the context provided, how can the risk of data breaches be mitigated? Under what circumstances would a medical institution choose to share information fully anonymously? Could a liability-sharing system alleviate medical institutions' reluctance to share information? What role does platform regulation play in the entire information chain, and are platforms willing to implement strict supervision for their own interests? Is it in the interest of insurance companies to implement an audit mechanism? How should government subsidy and penalty policies be coordinated to promote the development of medical data sharing?

According to extant literature, scholars have explored the utilization of digital technology to mitigate data breaches (3), while others have advocated for third-party oversight to prevent such incidents (4). In the context of medical data breaches, smart contracts and blockchains are utilized to enhance data sharing, thereby reducing the risk of leaks (5). Despite the reduced risk, medical institutions may struggle to absorb significant losses incurred from data breaches, thereby diminishing their enthusiasm for information sharing. This presents a novel challenge in the realm of medical data sharing. This paper applies evolutionary game theory to healthcare data sharing research, providing a theoretical framework to understand the dynamic interactions among stakeholders (e.g., medical institutions, data platforms, insurance companies). By constructing an evolutionary game model, we can analyze how each participant achieves a steady state through strategic adjustments amid data breach risks. This methodology is particularly suitable for exploring the choice of different strategies and their evolutionary trends in long-term cooperation and competition contexts (6). Previous research has seldom examined the role of medical data sharing in promoting the development of commercial health insurance and healthcare organizations. Section 2 verifies these gaps.

Building on this foundation, our study investigates the impact of medical data sharing on the growth of commercial health insurance and healthcare institutions, considering factors such as insurance claim efficiency, unreasonable charges, data breaches, and government reward and punishment policies. Within the context of digital platforms, this study examines a medical data information chain that includes medical institutions, digital platforms, and insurance companies. Medical institutions adopt strategies for anonymous information sharing, platforms implement regulatory measures, and insurance companies utilize auditing strategies. What factors influence the decision-making processes of these entities? Given the complex landscape of the digitalized medical industry data information chain involving multiple stakeholders, evolutionary game theory models can be employed to simulate and analyze the interactions and decision-making processes among the participants, thereby shedding light on their interrelationships. This paper investigates the strategies adopted by medical institutions, platforms, and insurance companies, delves into the choices made when these strategies reach equilibrium, and further explores how the information-sharing chain can enhance the performance of these three parties.

The study demonstrates that when healthcare organizations are responsible for data breaches, they tend to seek shared responsibility between platforms and insurers. This promotes stricter regulation by platforms and encourages insurers to conduct thorough audits. Conversely, if the platform or insurer is at fault, the liability-sharing system compels healthcare organizations to adopt a fully anonymous information-sharing strategy. Otherwise, they opt for partial anonymity to maximize benefits. In the event of a widespread data breach, compensation amounts increase, potentially overshadowing government supervision. Consequently, the government may impose penalties and reduce incentives for all involved parties.

The remainder of this paper is structured as follows: Section 2 provides a literature review and summarizes previous studies for comparative analysis with the current work. Section 3 outlines the problem description and basic assumptions. Section 4 develops the evolutionary game model. In Section 5, we conduct a numerical simulation analysis to validate the consistency of the simulation results with the theoretical derivations. Finally, Section 6 summarizes the findings and offers recommendations.

## 2 Literature review

### 2.1 Medical information sharing

The evolution of medical data information sharing has been ongoing for several years, currently moving toward digitalization, networking, and intelligentization. The establishment of inter-institutional information sharing among medical institutions facilitates the creation of regional medical information integration platforms, enabling the exchange and sharing of regional medical data across various platforms. This contributes to advancing medical reform, enhancing technological application, and improving social service capacities through the provision of solutions and strategies (7), thereby fostering an information-sharing network capable of reducing diagnostic error rates (8). The real-time sharing of patient information between medical institutions and patients can enhance the development of the healthcare industry and improve patient self-care (9). Doctors in medical settings can alleviate parental anxiety by sharing treatment details and actively involving parents in surgical decision-making processes (10). Notwithstanding advances in robust data anonymization techniques and the widely acknowledged advantages of data sharing, privacy concerns persist as a predominant obstacle to data exchange (11). Enhanced information transparency heightens risks for healthcare facilities; however, imposing caps on medical malpractice compensation may alleviate shortages in medical services caused by exorbitant claims, albeit this measure does not constitute a comprehensive remedy (12).

Based on our research, we find that few studies have shared medical data with insurance companies to promote the development of the insurance industry. We consider medical institutions, insurance companies, and platforms as game subjects and provide a theoretical framework to understand the dynamic interactions between these stakeholders (medical institutions, data platforms, and insurance companies). By constructing an evolutionary game model, we can analyze how each participant can achieve a stable state through strategic adjustments in the face of data breach risks.

### 2.2 Medical data breaches

Medical data breaches present a substantial challenge within the global data security domain. While patients and the public generally support data sharing for health research, they do so with certain conditions in place. Despite recognizing the potential benefits of data research, participants express apprehensions regarding data breaches (13). Scholars have investigated the integration of comprehensive privacy protection mechanisms within intelligent medical systems (14). The integration of emerging digital technologies in medical data management raises apprehensions regarding the potential introduction of novel inaccuracies and vulnerabilities (15). Following a data breach, medical institutions often switch partners, and the extent of the impact is dependent on the severity of the breach (16). Research has demonstrated that educating healthcare managers about risk factors can reduce the likelihood of data breaches (17). The integration of digital technology in establishing a network infrastructure significantly bolsters the security of healthcare information exchanges, consequently reducing the likelihood of healthcare data breaches (18).

To mitigate the risk of data breaches and enhance the willingness of medical institutions to share information, we integrate anonymous information-sharing strategies into their decision-making processes. We then examine the effects of these institutions' decisions regarding partially vs. fully anonymous information-sharing strategies on the overall supply chain.

### 2.3 Data-driven development of commercial health insurance

With the expansion of the insurance sector, commercial health insurance has become increasingly crucial as a beneficial supplementary tool in promoting residents' health. Some insurance companies even reduce their prices to stimulate market demand (19). Within this framework, the insurance sector necessitates more accurate data for product enhancements and demographic analysis (20). Accurate data can facilitate product optimization and enhance product credibility. By aligning product design more closely with residents' lifestyles, their willingness to purchase commercial health insurance can be increased (21). Improvements in credibility and economies of scale can lead to a reduction in insurance costs (22). Credit insurance mitigates the risk of retailer default, thereby facilitating manufacturers in augmenting their profitability through enhanced creditworthiness (23). Many insurance companies strive to enhance data precision through the advancement of digital technology. Nevertheless, the enhancement of digital capabilities does not significantly influence their adoption rates within these companies themselves (24).

This study reveals a gap in the literature concerning the factors influencing the information-sharing process and its effects within commercial health insurance. While existing research predominantly highlights the benefits of information sharing, there is scarce analysis on the determinants affecting this mechanism and the specific outcomes it impacts. We delve into aspects such as data breaches, reimbursement payments, penalty charges, subsidies, and unjustified fees to scrutinize their interplay with information sharing.

### 2.4 Government reward and penalty policies

Government reward and penalty policies are crucial for promoting the sharing of medical data. These policies aim to enhance the quality of medical services, standardize data-sharing practices, and protect patient privacy through various incentives and sanctions (25). These policies encompass fiscal subsidies (26), tax incentives (27), financial support (28), and punitive actions such as fines, administrative penalties, and market access restrictions (29). The operational details involve creating pertinent regulations, disseminating guiding principles, and establishing specific implementing agencies. For example, the government may grant financial rewards to medical institutions that proactively engage in data sharing and elevate service quality, while imposing fines or administrative penalties on those that breach data security protocols (30). Measures are designed to ensure that medical institutions adhere to laws and regulations while reaping the benefits of data-sharing efficiency, thereby preserving patients' privacy rights. In the event of a medical data breach, government reward and penalty policies can exert a positive influence (31).

Based on this premise, we find that while extensive literature exists on exploring and optimizing reward and punishment mechanisms, there is no precise mechanism tailored specifically for the healthcare information-sharing industry. We differentiate between penalty payments and compensation payments and delineate responsibilities for data breaches to promote the development of healthcare data and information sharing within the insurance industry.

## 3 Problem description and basic assumptions

### 3.1 Problem description

**Figure 1:**
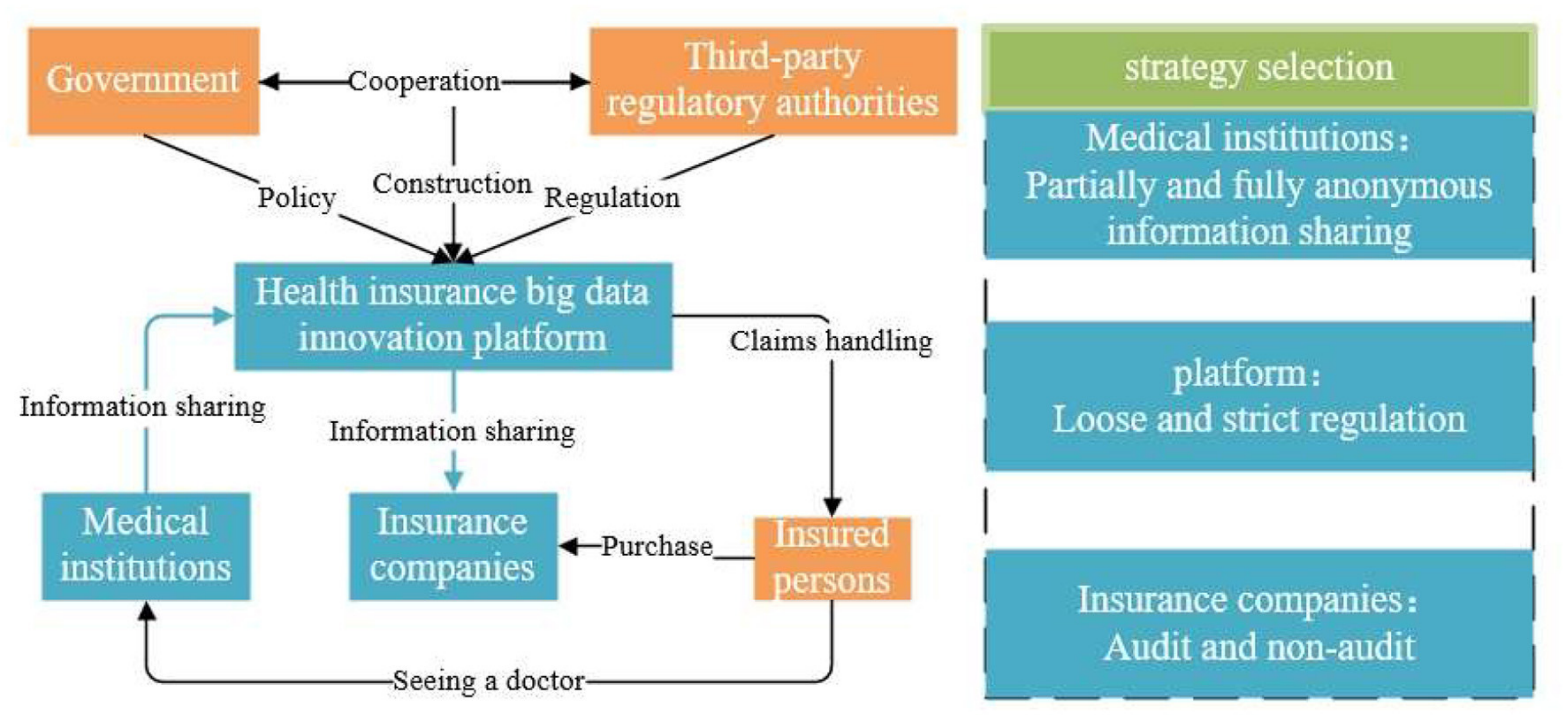
The process diagram of medical data information sharing and strategies for the three subjects.

As illustrated in Figure 1, the health insurance big data innovation platform is a government-led initiative. The government collaborates with a third-party regulator to establish and manage the platform. The government implements penalty and subsidy policies, while the third-party regulator oversees the operation and supervision of the platform. Insured individuals purchase commercial health insurance from insurance companies and can submit claims on the platform following medical consultations at healthcare institutions. Healthcare institutions share medical data with the platform, which in turn shares this data with insurance companies. This enables insurance firms to use the data more effectively for product innovation, actuarial pricing, and refined risk management. The strategic choices of healthcare institutions, the platform, and insurance companies within this three-party evolutionary game are depicted in Figure 1.

The strategy options for medical institutions, platforms, and insurance companies are illustrated in Figure 1. Medical institutions are obliged to protect patients' privacy and personal information security. By anonymizing the process, the risk of leaking sensitive information can be reduced to meet research or administrative needs. According to privacy protection theory, anonymization also achieves the principle of minimum necessity, i.e., collecting and processing only the necessary information required to accomplish a specific task, thereby reducing the risk of misuse or leakage of information. Therefore, the strategic choice for medical institutions is to partially or completely anonymize information sharing. Big data innovation platforms act as intermediaries to bridge the data flow between medical institutions and insurance companies. The regulatory mechanism is chosen not only to ensure the security and compliance of the data transfer process but also to establish a trust mechanism. According to governance theory, an effective regulatory mechanism can facilitate cooperation among participants and provide a reliable operational framework for data sharing. The existence of regulatory mechanisms helps platforms avoid potential legal risks and increases system transparency, thus boosting confidence among all stakeholders. Consequently, the strategic options for platforms are either strict or loose regulation. Insurance companies, as data users, need to balance the need for cost-benefit analysis and risk management when deciding whether to conduct an audit. According to risk management theory, audits are crucial tools for assessing data quality and integrity, aiding insurance companies in making more accurate risk assessments and pricing decisions. However, audits also introduce additional costs and technical complexity. Therefore, the strategic choice for insurance companies is whether to perform audit.

### 3.2 Basic assumptions

Table 1 presents the parameters and their descriptions. The specific hypotheses are outlined in Hypotheses 1 through 5 below.

**Table 1:** Parameter and descriptions.

**Parameter**	**Descriptions**	**Parameter**	**Descriptions**
*a*_1_	Probability of unreasonable charges	*n*_1_	Potential losses to medical institutions due to unreasonable charges
*f*_1_	Operating revenues of the platform	*c*_7_	Insurance companies need to give additional costs when medical institutions choose a partially anonymous strategy
*n*_2_	Insurance companies find unreasonable charges reduce cost losses	*f*_2_	Average benefit to insurance companies from claims efficiency
*u*_0_	Probability of insurance fraud occurring	*n*_0_	Fraudulent insurance coverage causes damage to insurance companies
*h*_2_	Rewards received by the platform for identifying security risks	*d*_0_	Compensation for victims of data breaches
*g*_1_	Proportion of compensation and fines borne by insurance companies	*g*_2_	Proportion of compensation and fines borne by the platform
1−*g*_1_−*g*_2_	Proportion of compensation and fines borne by insurance companies (0 ≤ *g*_1_+*g*_2_ < 1)	*p*_0_	The amount of fines imposed by the government after a data breach.
*m*_α_(*g*_α_)	Takes a value of 1 when *g*_α_ exceeds 0.5 and 0 otherwise		
The probability that a medical institution chooses a partially anonymous information sharing strategy is *x*		The probability that a medical institution chooses a completely anonymous information sharing strategy is 1−*x*	
*c*_2_	Information sharing pays off (*c*_1_<*c*_2_)	*c*_1_	Information sharing pays off
*b*_2_	Probability of not finding unreasonable charges (1>*b*_1_>*b*_2_>0)	*b*_1_	Probability of not finding unreasonable charges
*d*_6_	Probability of data breach in the information chain	*d*_5_	Probability of data breach in the information chain (0 < *d*_5_<*d*_6_ < 1)
*l*_2_	Amount of subsidy received	*l*_1_	Amount of subsidy received
The probability that the platform chooses a strict regulatory strategy is *y*		The probability that the platform chooses a loose regulatory strategy is 1−*y*	
*c*_4_	The cost of strict regulation	*c*_3_	The Costs of Loose Regulation
*d*_1_	Probability of finding problems such as safety hazards (0 < *d*_1_<*d*_2_ < 1)	*d*_2_	Probability of finding problems such as safety hazards
*d*_7_	Reduce the probability of a data breach occurring (*d*_7_<*d*_8_)	*d*_8_	Reduce the probability of a data breach occurring
The probability that the insurance company chooses the audit strategy is *z*		The probability that the insurance company chooses the no-audit strategy is 1−*z*	
*c*_6_	Cost of the audit	*c*_5_	Cost of not auditing
*d*_4_	Claims efficiency (0 < *d*_3_<*d*_4_)	*d*_3_	Claims efficiency
*b*_5_	Probability of not finding unreasonable charges in medical institutions (1>*b*_4_>*b*_5_>0)	*b*_4_	Probability of not finding unreasonable charges in medical institutions
$f_{2}\sin\left(\frac{\Pi }{2}d_{4}\right)$	Benefits from claims efficiency	$f_{2}\sin\left(\frac{\Pi }{2}d_{3}\right)$	Benefits from claims efficiency
*u*_2_	Probability of not detecting insurance fraud	*u*_1_	Probability of not detecting insurance fraud
*d*_8_	Reduce the probability of a data breach occurring		

Hypothesis 1: Medical data encompasses a vast amount of sensitive information, including medical history records and genetic test results. Leaks of such data can lead to severe consequences for patients and may result in legal liability and a loss of social trust for medical organizations. To mitigate these risks, medical institutions often opt for anonymized information sharing. However, the distinction between partial and complete anonymity influences both the benefits gained by the institutions and the likelihood of data breaches (32). Medical institutions choose between {Fully Anonymous Information Sharing, Partially Anonymous Information Sharing}. The probability of a medical institution choosing fully anonymous information sharing is 1−*x*, while the probability of choosing partially anonymous information sharing is *x*. Medical institutions choose to share information anonymously and obtain a profit of *c*_1_, while sharing partially anonymous information results in a profit of *c*_2_(*c*_1_<*c*_2_). The implementation of a fully anonymous information-sharing strategy can lead to diminished data traceability due to the complete anonymization of information. Consequently, this impairs the auditing and supervision of the medical service process, heightening the risk of unreasonable charges owing to the difficulty in tracing specific service items and detailed costs (33). With a probability of *a*_1_, unreasonable charges occur; if the medical institution fails to detect these unreasonable charges, which happens with a probability of *b*_1_, it incurs a potential loss of *n*_1_. When the medical institution partially shares information anonymously, the probability of failing to detect unreasonable charges is *b*_2_(1>*b*_1_>*b*_2_>0 ).

Hypothesis 2: The regulation of data platforms is crucial, as big data platforms grapple with the dual challenge of promoting data sharing while ensuring information security (34). Big Data Innovation Platform has two options: {Loose Regulation, Strict Regulation}. The probability of the platform choosing loose regulation is 1−*y*, and the probability of choosing strict regulation is *y*. The implementation of stringent regulatory policies necessitates the adoption of advanced security measures, encompassing but not limited to encryption technology, access control mechanisms, firewalls, and intrusion detection systems. These measures, while enhancing security, can significantly increase the operational costs of the platform. The cost for the platform to conduct loose supervision is *c*_3_, while the cost for strict supervision is *c*_4_(*c*_3_<*c*_4_). The data platform receives medical data shared by medical institutions and shares the processed data with insurance companies, and the platform receives operational revenues. The operational revenue of the platform is *f*_1_. Strict regulatory strategies are usually accompanied by more stringent security monitoring and preventive measures, which enable platforms to detect and respond to potential security threats earlier. Regular security reviews and testing also help to identify vulnerabilities and take remedial action in a timely manner. The probability of detecting safety risks and other issues under loose regulation is *d*_1_, whereas the probability under strict regulation is *d*_2_(0 < *d*_1_<*d*_2_ < 1 ).

Hypothesis 3: The implementation of audits by insurance companies is a key component in the process of sharing information between healthcare organizations and data platforms as well as insurance companies. It helps ensure the accuracy and security of data. For example, the Blue Cross Blue Shield Association and its member companies routinely conduct internal audits to ensure that their members are complying with health insurance-related laws, such as HIPAA. These audits include a review of data access controls, data integrity, and security in the EHR system. Insurance companies choose between {No Audit, Audit}. The probability of an insurance company opting for no audit is 1−*z*, and the probability of choosing to audit is *z*. The cost for an insurance company not auditing is *c*_5_, whereas the cost of auditing is *c*_6_. When medical institutions share information partially anonymously, resulting in increased data transparency, insurance companies incur an additional cost of *c*_7_. Insurance companies use data analytics tools to review medical bills to identify unusual charging patterns or potential fraud. These tools can process large amounts of data quickly and can compare it with historical data as well as data from other insurers to identify irregularities. Audits can also improve claims processing efficiency, and global insurers such as Allianz have been investing in technology solutions to improve the customer experience and increase operational efficiency. A patient's medical visit with unreasonable charges can cause the insurance company's claims to become larger, resulting in greater losses for the insurance company. After patients receive medical treatment and are discharged, they file for claims, with the efficiency of the process represented by a certain value, and the probability of not detecting unreasonable charges from the medical institution is also represented by a certain value. After an audit by the insurance company, the efficiency of the claims process is *d*_3_, and the probability of not detecting unreasonable charges is *b*_4_. If the insurance company discovers unreasonable charging practices, it will reduce the claim expenses by *d*_4_(0 < *d*_3_<*d*_4_). After an insurance company's claims efficiency has been improved to a certain extent, customer satisfaction may have reached a high level, and the effect of further efficiency improvements on customer satisfaction may gradually diminish (35). So we use the sin(*x*) function of $\left[0,\frac{\pi }{2}\right]$ to model the relationship between benefits and claims efficiency. Improvements in claims efficiency attract consumers to purchase insurance products, with the efficiency affecting the revenue through a function represented by $f=f_{2}\sin\left(\frac{\Pi }{2}d_{i}\right)$ (*i* = 3, 4), and *f*_2_ represents the average revenue brought about by improvements in claims efficiency. The probability of fraudulent claims is *u*_0_; the probability of not being detected when the insurance company does not audit is *u*_1_; the probability of not being detected when the insurance company does audit is *u*_2_(1>*u*_1_>*u*_2_>0), and the loss caused by fraudulent claims is *n*_0_.

Hypothesis 4: Fully anonymous information sharing strategy protects patient privacy and can reduce the risk of data leakage when sharing information. The risk of information leakage is higher for partially anonymized information sharing strategy than the former. When medical institutions engage in fully anonymous information sharing, the probability of data breaches in the information chain is *d*_5_. When partially sharing information anonymously, the probability of a data breach occurring is *d*_6_(0 < *d*_5_<*d*_6_ < 1). When a medical institution chooses a partially or fully anonymous information sharing strategy, the risk of information breach due to incomplete masking of information is higher for partially anonymous information sharing than for fully anonymous information sharing. Platforms with strictly regulated strategies will use more secure protection measures to reduce the probability of data breach. Insurance companies monitor, process, and store large amounts of data by means of auditing to achieve a reduction in the probability of data breaches. Strict regulation by the platform reduces the probability of a data breach by *d*_7_, and audits implemented by the insurance company also reduce the probability of a data breach by *d*_8_. If the breach violates relevant laws and regulations (e.g., GDPR, HIPAA, etc.), individual subjects can face fines. Subjects may be required to provide credit monitoring services or other forms of compensation to affected customers. Data breaches lead to a decrease in public and customer trust in a business, which can affect future sales and market share. When a data breach occurs, all three subjects experience indirect and direct losses. The responsible parties are required to provide compensation to the victims, amounting to *d*_0_. The medical institution bears a proportion of *g*_1_, third-party organizations bear a proportion of *g*_2_, and the insurance company bears a proportion of 1−*g*_1_−*g*_2_(0 ≤ *g*_1_+*g*_2_ < 1 ).

Hypothesis 5: In the process of constructing information sharing between medical institutions and data platforms as well as insurance companies, the China Health Insurance Bureau mentioned that the risk of information leakage would make each subject participate in information sharing negatively, so it proposed to share the risk of leakage and impose penalties in case of leakage. In the event of a data breach, the government penalizes members of the information chain, with each subject bearing a different percentage of the penalty. The government also promotes anonymous information sharing among healthcare providers in the form of subsidies, as in the case of Jining City, China, which issued the Implementing Opinions on Further Improving and Perfecting the Mechanism for Sharing Information and Data on Persons Receiving Medical Assistance, which aims to further improve the mechanism for sharing information and data on medical assistance recipients. The government policy is divided into subsidy policy and penalty policy. Penalty policy: the function of penalty payment is *p*_0_*m*_α_(*g*_α_)(α = 1, 2, 3), *p*_0_ is the amount of the fine, *m*_α_(*g*_α_) is an indicator function that takes the value of 1 when *g*_α_exceeds 0.5, and 0 otherwise. If *m*_*a*_(*g*_*a*_) in *g*_*a*_ are < 0.5, it means that it is impossible to find out which subject caused the data breach, at this time, medical institutions, insurance companies and platforms only need to pay compensation to the patients do not need to pay penalties. Subsidy policy: the amount of subsidy under a fully anonymous information sharing policy for a medical institution is*l*_1_, and the amount of subsidy under a partially anonymous information sharing policy is *l*_2_(*l*_1_>*l*_2_). The platforms are rewarded for identifying problems in the information chain such as data security risks *h*_2_.

## 4 Responsibility sharing model

### 4.1 Model construction

Based on the aforementioned assumptions of this model, an evolutionary game model has been established. The payoff matrices for medical institutions, platform, and insurance companies are presented in Tables 2, 3. The probability that a medical institution chooses a partially anonymous information sharing strategy is x, and the probability that a medical institution chooses a fully anonymous information sharing strategy is 1 – *x*; the probability that a big data innovation platform chooses a strictly regulated strategy is *y*, and the probability that a big data innovation platform chooses a loose regulated strategy is 1 – *y*. The probability that an insurance company chooses an auditing strategy is *z*, and the probability that an insurance company chooses a no-auditing strategy is 1 – *z*. Revenue components of medical institutions: revenue from implementing strategies + subsidies – compensation payments – penalties – losses due to unreasonable fees; revenue components of platforms: operational revenue + rewards for detecting safety hazards – compensation payments – penalties – costs of implementing strategies; revenue components of insurance companies: revenue from claims efficiency – costs of implementing strategies – losses due to unreasonable fees – compensation payments – penalties – losses due to fraudulent insurance policies Losses due to insurance fraud.

**Table 2:** Three-party payoff matrix under strict regulation (*y*) by the platform.

**Game participants**		**Medical institutions**	
		**Audit** (**z**)	**Non-audit** (**1−*z***)
Insurance companies	Partially anonymous information sharing (*x*)	*c*_2_−*a*_1_*b*_2_*b*_5_*n*_1_−*d*_6_*d*_7_*d*_8_(*g*_1_*d*_0_+*p*_0_*m*_1_)+*l*_2_ *f*_1_−*c*_4_+*d*_2_*h*_2_−*d*_6_*d*_7_*d*_8_(*g*_2_*d*_0_+*p*_0_*m*_2_) $f_{2}\sin\left(\frac{\Pi }{2}d_{4}\right)-c_{6}-c_{7}-a_{1}b_{2}b_{5}n_{2}-u_{0}u_{2}n_{0}-$*d*_6_*d*_7_*d*_8_[(1−*g*_1_−*g*_2_)*d*_0_+*p*_0_*m*_3_]
	*c*_2_−*a*_1_*b*_2_*b*_4_*n*_1_−*d*_6_*d*_7_(*g*_1_*d*_0_+*p*_0_*m*_1_)+*l*_2_ *f*_1_−*c*_4_+*d*_2_*h*_2_−*d*_6_*d*_7_(*g*_2_*d*_0_+*p*_0_*m*_2_) $f_{2}\sin\left(\frac{\Pi }{2}d_{3}\right)-c_{5}-c_{7}-a_{1}b_{2}b_{4}n_{2}-u_{0}u_{1}n_{0}-$*d*_6_*d*_7_[(1−*g*_1_−*g*_2_)*d*_0_+*p*_0_*m*_3_]
	Fully anonymous information sharing (1−*x*)	*c*_1_−*a*_1_*b*_1_*b*_5_*n*_1_−*d*_5_*d*_7_*d*_8_(*g*_1_*d*_0_+*p*_0_*m*_1_)+*l*_1_ *f*_1_−*c*_4_+*d*_2_*h*_2_−*d*_5_*d*_7_*d*_8_(*g*_2_*d*_0_+*p*_0_*m*_2_) $f_{2}\sin\left(\frac{\Pi }{2}d_{4}\right)-c_{6}-a_{1}b_{1}b_{5}n_{2}-u_{0}u_{2}n_{0}-$
*d*_5_*d*_7_*d*_8_[(1−*g*_1_−*g*_2_)*d*_0_+*p*_0_*m*_3_]
	*c*_1_−*a*_1_*b*_1_*b*_4_*n*_1_−*d*_5_*d*_7_(*g*_1_*d*_0_+*p*_0_*m*_1_)+*l*_1_ *f*_1_−*c*_4_+*d*_2_*h*_2_−*d*_5_*d*_7_(*g*_2_*d*_0_+*p*_0_*m*_2_) $f_{2}\sin\left(\frac{\Pi }{2}d_{3}\right)-c_{5}-a_{1}b_{1}b_{4}n_{2}-u_{0}u_{1}n_{0}-$*d*_5_*d*_7_[(1−*g*_1_−*g*_2_)*d*_0_+*p*_0_*m*_3_]

**Table 3:** Three-party payoff matrix under loose regulation (1−*y*) by the platform.

**Game participants**		**Medical institutions**	
		**Audit** (**z**)	**Non-audit** (**1−*z***)
Insurance companies	Partially anonymous information sharing (*x*)	*c*_2_−*a*_1_*b*_2_*b*_5_*n*_1_−*d*_6_*d*_8_(*g*_1_*d*_0_+*p*_0_*m*_1_)+*l*_2_ *f*_1_−*c*_3_+*d*_1_*h*_2_−*d*_6_*d*_8_(*g*_2_*d*_0_+*p*_0_*m*_2_) $f_{2}\sin\left(\frac{\Pi }{2}d_{4}\right)-c_{6}-c_{7}-a_{1}b_{2}b_{5}n_{2}-u_{0}u_{2}n_{0}-$*d*_6_*d*_8_[(1−*g*_1_−*g*_2_)*d*_0_+*p*_0_*m*_3_]
	*c*_2_−*a*_1_*b*_2_*b*_4_*n*_1_−*d*_6_(*g*_1_*d*_0_+*p*_0_*m*_1_)+*l*_2_ *f*_1_−*c*_3_+*d*_1_*h*_2_−*d*_6_(*g*_2_*d*_0_+*p*_0_*m*_2_) $f_{2}\sin\left(\frac{\Pi }{2}d_{3}\right)-c_{5}-c_{7}-a_{1}b_{2}b_{4}n_{2}-u_{0}u_{1}n_{0}-$*d*_6_[(1−*g*_1_−*g*_2_)*d*_0_+*p*_0_*m*_3_]
	Fully anonymous information sharing (1−*x*)	*c*_1_−*a*_1_*b*_1_*b*_5_*n*_1_−*d*_5_*d*_8_(*g*_1_*d*_0_+*p*_0_*m*_1_)+*l*_1_ *f*_1_−*c*_3_+*d*_1_*h*_2_−*d*_5_*d*_8_(*g*_2_*d*_0_+*p*_0_*m*_2_) $f_{2}\sin\left(\frac{\Pi }{2}d_{4}\right)-c_{6}-a_{1}b_{1}b_{5}n_{2}-u_{0}u_{2}n_{0}-$*d*_5_*d*_8_[(1−*g*_1_−*g*_2_)*d*_0_+*p*_0_*m*_3_]
	*c*_1_−*a*_1_*b*_1_*b*_4_*n*_1_−*d*_5_(*g*_1_*d*_0_+*p*_0_*m*_1_)+*l*_1_ *f*_1_−*c*_3_+*d*_1_*h*_2_−*d*_5_(*g*_2_*d*_0_+*p*_0_*m*_2_) $f_{2}\sin\left(\frac{\Pi }{2}d_{3}\right)-c_{5}-a_{1}b_{1}b_{4}n_{2}-u_{0}u_{1}n_{0}-$*d*_5_[(1−*g*_1_−*g*_2_)*d*_0_+*p*_0_*m*_3_]

### 4.2 Replicating dynamic equations and evolutionary equilibria

#### 4.2.1 Strategic stability analysis of medical organizations

In Tables 2, 3, there are benefit matrices that represent the medical institutions. Based on the payoff matrix constructed in the previous text, the expected revenue for medical institutions under partially anonymous information sharing is obtained:


(1) $$\begin{array}{l} \begin{array}{l} E_{A1}=yz\left\{c_{2}-a_{1}b_{2}b_{5}n_{1}-d_{6}d_{7}d_{8}\left(g_{1}d_{0}+p_{0}m_{1}\right)+l_{2}\right\}\\ +y\left(1-z\right)\left\{c_{2}-a_{1}b_{2}b_{4}n_{1}-d_{6}d_{7}\left(g_{1}d_{0}+p_{0}m_{1}\right)+l_{2}\right\}\\ +\left(1-y\right)z\left\{c_{2}-a_{1}b_{2}b_{5}n_{1}-d_{6}d_{8}\left(g_{1}d_{0}+p_{0}m_{1}\right)+l_{2}\right\}\\ +\left(1-y\right)\left(1-z\right)\left\{c_{2}-a_{1}b_{2}b_{4}n_{1}-d_{6}\left(g_{1}d_{0}+p_{0}m_{1}\right)+l_{2}\right\} \end{array} \end{array}$$

Expected benefits of fully anonymous information sharing for medical institutions:


(2) $$\begin{array}{l} \begin{array}{l} E_{A2}=yz\left\{c_{1}-a_{1}b_{1}b_{5}n_{1}-d_{5}d_{7}d_{8}\left(g_{1}d_{0}+p_{0}m_{1}\right)+l_{1}\right\}\\ +y\left(1-z\right)\left\{c_{1}-a_{1}b_{1}b_{4}n_{1}-d_{5}d_{7}\left(g_{1}d_{0}+p_{0}m_{1}\right)+l_{1}\right\}\\ +\left(1-y\right)z\left\{c_{1}-a_{1}b_{1}b_{5}n_{1}-d_{5}d_{8}\left(g_{1}d_{0}+p_{0}m_{1}\right)+l_{1}\right\}\\ +\left(1-y\right)\left(1-z\right)\left\{c_{1}-a_{1}b_{1}b_{4}n_{1}-d_{5}\left(g_{1}d_{0}+p_{0}m_{1}\right)+l_{1}\right\} \end{array} \end{array}$$

The average expected return for medical institutions is:


(3) $$\begin{array}{l} E_{A}=xE_{A1}+\left(1-x\right)E_{A2} \end{array}$$

From Equations 1–3, the equation for the replication dynamics of the medical institution is given as:


(4) $$\begin{array}{l} \begin{array}{l} F_{x}\left(x\right)=\frac{dx}{dt}=x\left(1-x\right)\left(E_{A1}-E_{A2}\right)\\ =x\left(1-x\right)\{yz[c_{2}-c_{1}-a_{1}b_{5}n_{1}\left(b_{2}-b_{1}\right)-d_{7}d_{8}\left(g_{1}d_{0}\right)\\ \left(d_{6}-d_{5}\right)-d_{7}d_{8}p_{0}m_{1}\left(d_{6}p_{2}-d_{5})+l_{2}-l_{1}\right]+\\ y\left(1-z\right)[c_{2}-c_{1}-a_{1}b_{4}n_{1}\left(b_{2}-b_{1}\right)-d_{7}\left(g_{1}d_{0}\right)\\ \left(d_{6}-d_{5}\right)-d_{7}p_{0}m_{1}\left(d_{6}p_{2}-d_{5})+l_{2}-l_{1}\right]+\\ \left(1-y\right)z[c_{2}-c_{1}-a_{1}b_{5}n_{1}\left(b_{2}-b_{1}\right)-d_{8}\left(g_{1}d_{0}\right)\left(d_{6}-d_{5}\right)\\ -d_{8}p_{0}m_{1}\left(d_{6}p_{2}-d_{5})+l_{2}-l_{1}\right]+\\ \left(1-y\right)\left(1-z\right)[c_{2}-c_{1}-a_{1}b_{4}n_{1}\left(b_{2}-b_{1}\right)-\left(g_{1}d_{0}\right)\left(d_{6}-d_{5}\right)\\ -p_{0}m_{1}\left(d_{6}p_{2}-d_{5})+l_{2}-l_{1}\right]\} \end{array} \end{array}$$

#### 4.2.2 Strategic stability analysis of digital platforms

In Tables 2, 3, there are benefit matrices that represent the platform. With the benefit matrix constructed above, the expected benefit of strict regulation of the platform can be obtained as:


(5) $$\begin{array}{l} \begin{array}{l} E_{B1}=xz\left\{f_{1}-c_{4}+d_{2}h_{2}-d_{6}d_{7}d_{8}\left(g_{2}d_{0}+p_{0}m_{2}\right)\right\}\\ +x\left(1-z\right)\left\{f_{1}-c_{4}+d_{2}h_{2}-d_{6}d_{7}\left(g_{2}d_{0}+p_{0}m_{2}\right)\right\}\\ +\left(1-x\right)z\left\{f_{1}-c_{4}+d_{2}h_{2}-d_{5}d_{7}d_{8}\left(g_{2}d_{0}+p_{0}m_{2}\right)\right\}\\ +\left(1-x\right)\left(1-z\right)\left\{f_{1}-c_{4}+d_{2}h_{2}-d_{5}d_{7}\left(g_{2}d_{0}+p_{0}m_{2}\right)\right\} \end{array} \end{array}$$

The expected return on the platform's loose regulation is:


(6) $$\begin{array}{l} \begin{array}{l} E_{B2}=xz\left\{f_{1}-c_{3}+d_{1}h_{2}-d_{6}d_{8}\left(g_{2}d_{0}+p_{0}m_{2}\right)\right\}\\ +x\left(1-z\right)\left\{f_{1}-c_{3}+d_{1}h_{2}-d_{6}\left(g_{2}d_{0}+p_{0}m_{2}\right)\right\}\\ +\left(1-x\right)z\left\{f_{1}-c_{3}+d_{1}h_{2}-d_{5}d_{8}\left(g_{2}d_{0}+p_{0}m_{2}\right)\right\}\\ +\left(1-x\right)\left(1-z\right)\left\{f_{1}-c_{3}+d_{1}h_{2}-d_{5}\left(g_{2}d_{0}+p_{0}m_{2}\right)\right\} \end{array} \end{array}$$

The average expected return of the platform is.


(7) $$\begin{array}{l} E_{B}=yE_{B1}+\left(1-y\right)E_{B2} \end{array}$$

From Equations 5–7, the replicated dynamic equation of the platform is given as:


(8) $$\begin{array}{l} \begin{array}{c} F_{y}\left(y\right)=\frac{dy}{dt}=y\left(1-y\right)\left(E_{B1}-E_{B2}\right)\\ =\{xz[c_{3}-c_{4}+\left(d_{2}-d_{1}\right)h_{2}-d_{6}d_{8}\left(g_{2}d_{0}+n_{4}-p_{0}m_{2}\right)\\ \left(d_{7}-1)\right]+\\ x\left(1-z\right)[c_{3}-c_{4}+\left(d_{2}-d_{1}\right)h_{2}-d_{6}\left(g_{2}d_{0}+n_{4}-p_{0}m_{2}\right)\\ \left(d_{7}-1)\right]+\\ \left(1-x\right)z[c_{3}-c_{4}+\left(d_{2}-d_{1}\right)h_{2}-d_{5}d_{8}\left(g_{2}d_{0}+n_{4}-p_{0}m_{2}\right)\\ \left(d_{7}-1)\right]+\\ \left(1-x\right)\left(1-z\right)[c_{3}-c_{4}+\left(d_{2}-d_{1}\right)h_{2}\\ -d_{5}(g_{2}d_{0}+n_{4}-p_{0}m_{2})\left(d_{7}-1)\right]\} \end{array} \end{array}$$

#### 4.2.3 Strategic stability analysis of insurance companies

In Tables 2, 3, there are benefit matrices that represent the insurance company. With the benefit matrix constructed above, the expected benefit of the insurance company audit is obtained as:


(9) $$\begin{array}{l} \begin{array}{l} E_{C1}=xy\{f_{2}\sin\left(\frac{\text{Π}}{2}d_{4}\right)-c_{6}-c_{7}-a_{1}b_{2}b_{5}n_{2}-u_{0}u_{2}n_{0}\\ -d_{6}d_{7}d_{8}\left[\left(1-g_{1}-g_{2})d_{0}+p_{0}m_{3}\right]\right\}\\ +x\left(1-y\right)\{f_{2}\sin\left(\frac{\text{Π}}{2}d_{4}\right)-c_{6}-c_{7}-a_{1}b_{2}b_{5}n_{2}-u_{0}u_{2}n_{0}\\ -d_{6}d_{8}\left[\left(1-g_{1}-g_{2}\right)d_{0}+p_{0}m_{3}\right]\}\\ +\left(1-x\right)y\{f_{2}\sin\left(\frac{\text{Π}}{2}d_{4}\right)-c_{6}-a_{1}b_{1}b_{5}n_{2}-u_{0}u_{2}n_{0}\\ -d_{5}d_{7}d_{8}[\left(1-g_{1}-g_{2}\right)d_{0}+p_{0}m_{3}]\}\\ +\left(1-x\right)\left(1-y\right)\{f_{2}\sin\left(\frac{\text{Π}}{2}d_{4}\right)-c_{6}-a_{1}b_{1}b_{5}n_{2}-u_{0}u_{2}n_{0}\\ -d_{5}d_{8}\left[\left(1-g_{1}-g_{2})d_{0}+n_{5}+p_{0}m_{3}\right]\right\} \end{array} \end{array}$$

The expected return on an insurance company's non-audit is:


(10) $$\begin{array}{l} \begin{array}{l} E_{C2}=xy\{f_{2}\sin\left(\frac{\text{Π}}{2}d_{3}\right)-c_{5}-c_{7}-a_{1}b_{2}b_{4}n_{2}-u_{0}u_{1}n_{0}\\ -d_{6}d_{7}\left[\left(1-g_{1}-g_{2})d_{0}+p_{0}m_{3}\right]\right\}\\ +x\left(1-y\right)\{f_{2}\sin\left(\frac{\text{Π}}{2}d_{3}\right)-c_{5}-c_{7}-a_{1}b_{2}b_{4}n_{2}-u_{0}u_{1}n_{0}\\ -d_{6}\left[\left(1-g_{1}-g_{2})d_{0}+p_{0}m_{3}\right]\right\}\\ +\left(1-x\right)y\{f_{2}\sin\left(\frac{\text{Π}}{2}d_{3}\right)-c_{5}-a_{1}b_{1}b_{4}n_{2}-u_{0}u_{1}n_{0}\\ -d_{5}d_{7}\left[\left(1-g_{1}-g_{2})d_{0}+p_{0}m_{3}\right]\right\}\\ +\left(1-x\right)\left(1-y\right)\{f_{2}\sin\left(\frac{\text{Π}}{2}d_{3}\right)-c_{5}-a_{1}b_{1}b_{4}n_{2}-u_{0}u_{1}n_{0}\\ -d_{5}\left[\left(1-g_{1}-g_{2})d_{0}+n_{5}+p_{0}m_{3}\right]\right\} \end{array} \end{array}$$

From Equations 9–11, the replicated dynamic equation for the insurance company is given as:


(11) $$\begin{array}{l} \begin{array}{l} F_{z}\left(z\right)=\frac{dz}{dt}=z\left(1-z\right)\left(E_{C1}-E_{C2}\right)\\ =\langle xy\{f_{2}[\sin\left(\frac{\text{Π}}{2}d_{4}\right)-\sin(\frac{\text{Π}}{2}d_{3})]-c_{6}+c_{5}-a_{1}b_{2}n_{2}(b_{5}-b_{4})\\ -u_{0}n_{0}\left(u_{2}-u_{1}\right)-d_{6}d_{7}\left[\left(1-g_{1}-g_{2}\right)d_{0}+n_{5}-p_{0}m_{3}\right]\\ (d_{8}-1)\}+\\ x\left(1-y\right)\{f_{2}\left[\sin\left(\frac{\text{Π}}{2}d_{4}\right)-\sin\left(\frac{\text{Π}}{2}d_{3}\right)\right]-c_{6}+c_{5}\\ -a_{1}b_{2}n_{2}\left(b_{5}-b_{4}\right)-u_{0}n_{0}\left(u_{2}-u_{1}\right)-d_{6}[\left(1-g_{1}-g_{2}\right)d_{0}\\ +n_{5}-p_{0}m_{3}](d_{8}-1)\}+\\ \left(1-x\right)y\{f_{2}\left[\sin\left(\frac{\text{Π}}{2}d_{4}\right)-\sin\left(\frac{\text{Π}}{2}d_{3}\right)\right]-c_{6}+c_{5}\\ -a_{1}b_{2}n_{2}\left(b_{5}-b_{4}\right)-u_{0}n_{0}\left(u_{2}-u_{1}\right)-d_{5}d_{7}[\left(1-g_{1}-g_{2}\right)d_{0}\\ +n_{5}-p_{0}m_{3}](d_{8}-1)\}+\\ \left(1-x\right)\left(1-y\right)\{f_{2}\left[\sin\left(\frac{\text{Π}}{2}d_{4}\right)-\sin\left(\frac{\text{Π}}{2}d_{3}\right)\right]-c_{6}+c_{5}\\ -a_{1}b_{2}n_{2}\left(b_{5}-b_{4}\right)-u_{0}n_{0}\left(u_{2}-u_{1}\right)-d_{5}[\left(1-g_{1}-g_{2}\right)d_{0}\\ +n_{5}-p_{0}m_{3}](d_{8}-1)\}\rangle \end{array} \end{array}$$

### 4.3 Analysis of stability and evolutionary results of the system

#### 4.3.1 Stability analysis of the equilibrium point

Based on the previously mentioned chapter on the replicator dynamic equations [*F*_*x*_(*x*), *F*_*y*_(*y*), *F*_*z*_(*z*)] of the three parties in the game, we can further obtain the Jacobian matrix of the evolutionary game system under the responsibility sharing mode.


$$\begin{array}{l} J=\left[\begin{array}{ccc} \frac{\partial F_{x}\left(x\right)}{\partial x} & \frac{\partial F_{x}\left(x\right)}{\partial y} & \frac{\partial F_{x}\left(x\right)}{\partial z}\\ \frac{\partial F_{y}\left(y\right)}{\partial x} & \frac{\partial F_{y}\left(y\right)}{\partial y} & \frac{\partial F_{y}\left(y\right)}{\partial z}\\ \frac{\partial F_{z}\left(z\right)}{\partial x} & \frac{\partial F_{z}\left(z\right)}{\partial y} & \frac{\partial F_{z}\left(z\right)}{\partial z} \end{array}\right]\; \end{array}$$

According to the Liapunov system stability criterion, the equilibrium is asymptotically stable when all eigenvalues of the Jacobi matrix satisfy the condition; if one or more λ < 0, then the equilibrium is unstable λ>0. By setting *F*_*x*_(*x*) = 0, *F*_*y*_(*y*) = 0, and *F*_*z*_(*z*) = 0, we can obtain 8 local equilibrium points: *E*_1_ = (0, 0, 0), *E*_2_ = (0, 0, 1), *E*_3_ = (0, 1, 0), *E*_4_ = (1, 0, 0), *E*_5_ = (1, 1, 0), *E*_6_ = (1, 0, 1), *E*_7_ = (0, 1, 1), *E*_8_ = (1, 1, 1), where the elements in parentheses correspond to the values of strategies (*x, y, z*) to be adopted by the medical institution, platform, and insurance company at each equilibrium point, respectively. According to the Lyapunov theorem, when all the eigenvalues of the Jacobian matrix are negative, the local equilibrium point is an Evolutionarily Stable Strategy (ESS). The eigenvalues of the Jacobian matrix corresponding to each equilibrium point are calculated as shown in Table 4.

**Table 4:** Stability analysis of equilibrium points.

**Equilibrium point**	**Matrix eigenvalues**			**Stability**
	**λ**_**1**_	**λ**_**2**_	**λ**_**3**_	
*E*_1_(0, 0, 0)	*a*_1_*b*_4_*n*_1_(*b*_1_−*b*_2_)+*c*_2_−*c*_1_+ (*d*_0_*g*_1_+*m*_1_*p*_0_)(*d*_5_−*d*_6_)+ *l*_2_−*l*_1_	*c*_3_−*c*_4_+*d*_0_*d*_5_*g*_2_(1−*d*_7_)+ (*d*_2_−*d*_1_)*h*_2_+*d*_5_*m*_2_*p*_0_(1−*p*_4_)	*a*_1_*b*_1_*n*_2_(*b*_4_−*b*_5_)+*c*_5_−*c*_6_+*d*_0_*d*_5_*g*_1_(*d*_8_−1) +*d*_0_*d*_5_*g*_2_(*d*_8_−1)+*d*_0_*d*_5_(1−*d*_8_)+ $d_{5}m_{3}p_{0}\left(1-d_{8}\right)+f_{2}\left[\sin\left(\frac{\Pi d_{4}}{2}\right)-\sin\left(\frac{\Pi d_{3}}{2}\right)\right]+$ *n*_0_*u*_0_(*u*_1_−*u*_2_)	(*, *, *)
*E*_2_(0, 0, 1)	*a*_1_*b*_5_*n*_1_(*b*_1_−*b*_2_)+*c*_2_−*c*_1_+ (*d*_0_*d*_8_*g*_1_+*d*_8_*m*_1_*p*_0_)(*d*_5_−*d*_6_)+ *l*_2_−*l*_1_	*c*_3_−*c*_4_+*d*_0_*d*_5_*d*_8_*g*_2_(1−*d*_7_)+ (*d*_2_−*d*_1_)*h*_2_+ *d*_5_*d*_8_*m*_2_*p*_0_(1−*d*_7_)	*a*_1_*b*_2_*n*_2_(*b*_5_−*b*_4_)+*c*_6_−*c*_5_+*d*_0_*d*_5_*g*_1_(1−*d*_8_) +*d*_0_*d*_5_*g*_2_(1−*d*_8_)+*d*_0_*d*_5_(*d*_8_−1) +*d*_5_*m*_3_*p*_0_(*d*_8_−1)+ $f_{2}\left[\sin\left(\frac{\Pi d_{3}}{2}\right)-\sin\left(\frac{\Pi d_{4}}{2}\right)\right]+$ *n*_0_*u*_0_(*u*_2_−*u*_1_)	(*, *, *)
*E*_3_(0, 1, 0)	*a*_1_*b*_4_*n*_1_(*b*_1_−*b*_2_)+*c*_2_−*c*_1_+ (*d*_0_*d*_7_*g*_1_+*d*_7_*m*_1_*p*_0_)(*d*_5_−*d*_6_)+ *l*_2_−*l*_1_	*c*_4_−*c*_3_+*d*_0_*d*_5_*g*_2_(*d*_7_−1)+ (*d*_1_−*d*_2_)*h*_2_+*d*_5_*m*_2_*p*_0_(*d*_7_−1)	*a*_1_*b*_2_*n*_2_(*b*_4_−*b*_5_)+*c*_5_−*c*_6_+*d*_0_*d*_5_*d*_7_*g*_1_(*d*_8_−1) +*d*_0_*d*_5_*d*_7_*g*_2_(*d*_8_−1)+*d*_0_*d*_5_*d*_7_(1−*d*_8_) +*d*_5_*d*_7_*m*_3_*p*_0_(1−*d*_8_) $+f_{2}\left[\sin\left(\frac{\Pi d_{4}}{2}\right)-\sin\left(\frac{\Pi d_{3}}{2}\right)\right]+$ *n*_0_*u*_0_(*u*_1_−*u*_2_)	(*, *, *)
*E*_4_(1, 0, 0)	*a*_1_*b*_4_*n*_1_(*b*_2_−*b*_1_)+*c*_1_−*c*_2_+ (*d*_0_*g*_1_+*m*_1_*p*_0_)(*d*_6_−*d*_5_)+ *l*_1_−*l*_2_	*c*_3_−*c*_4_+*d*_0_*d*_6_*g*_2_(1−*d*_7_)+ (*d*_2_−*d*_1_)*h*_2_+ *d*_6_*m*_2_*p*_0_(1−*d*_7_)	*a*_1_*b*_2_*n*_2_(*b*_4_−*b*_5_)+*c*_5_−*c*_6_+*d*_0_*d*_6_*g*_1_(*d*_8_−1) +*d*_0_*d*_6_*g*_2_(*d*_8_−1)+*d*_0_*d*_6_(1−*d*_8_) $+d_{6}m_{3}p_{0}\left(1-p_{6}\right)+f_{2}\left[\sin\left(\frac{\Pi d_{4}}{2}\right)-\sin\left(\frac{\Pi d_{3}}{2}\right)\right]$ +*n*_0_*u*_0_(*u*_1_−*u*_2_)	(*, *, *)
*E*_5_(1, 1, 0)	*a*_1_*b*_4_*n*_1_(*b*_2_−*b*_1_)+*c*_1_−*c*_2_+ (*d*_0_*d*_7_*g*_1_+*d*_7_*m*_1_*p*_0_)(*d*_6_−*d*_5_)+ *l*_1_−*l*_2_	*c*_4_−*c*_3_+*d*_0_*d*_6_*g*_2_(*d*_7_−1)+ (*d*_1_−*d*_2_)*h*_2_+*d*_6_*m*_2_*p*_0_(*d*_7_−1)	*a*_1_*b*_2_*n*_2_(*b*_4_−*b*_5_)+*c*_5_−*c*_6_+*d*_0_*d*_6_*d*_7_*g*_1_(*d*_8_−1) +*d*_0_*d*_6_*d*_7_*g*_2_(*d*_8_−1)+*d*_0_*d*_6_*d*_7_(1−*d*_8_) +*d*_6_*d*_7_*m*_3_*p*_0_(1−*d*_8_) $+f_{2}\left[\sin\left(\frac{\Pi d_{4}}{2}\right)-\sin\left(\frac{\Pi d_{3}}{2}\right)\right]+$ *n*_0_*u*_0_(*u*_1_−*u*_2_)	(*, *, *)
*E*_6_(1, 0, 1)	*a*_1_*b*_5_*n*_1_(*b*_2_−*b*_1_)+*c*_1_−*c*_2_+ (*d*_0_*d*_8_*g*_1_+*d*_8_*m*_1_*p*_0_)(*d*_6_−*d*_5_)+ *l*_1_−*l*_2_	*c*_3_−*c*_4_+*d*_0_*d*_6_*d*_8_*g*_2_(1−*d*_7_)+ (*d*_2_−*d*_1_)*h*_2_+*d*_6_*d*_8_*m*_2_*p*_0_(1−*d*_7_)	*a*_1_*b*_2_*n*_2_(*b*_5_−*b*_4_)+*c*_6_−*c*_5_+*d*_0_*d*_6_*g*_1_(1−*d*_8_) +*d*_0_*d*_6_*g*_2_(1−*d*_8_)+*d*_0_*d*_6_(*d*_8_−1) +*d*_6_*m*_3_*p*_0_(*d*_8_−1) $+f_{2}\left[\sin\left(\frac{\Pi d_{3}}{2}\right)-\sin\left(\frac{\Pi d_{4}}{2}\right)\right]$ +*n*_0_*u*_0_(*u*_2_−*u*_1_)	(*, *, *)
*E*_7_(0, 1, 1)	*a*_1_*b*_5_*n*_1_(*b*_1_−*b*_2_)+*c*_2_−*c*_1_+ (*d*_0_*d*_7_*d*_8_*g*_1_+*d*_7_*d*_8_*m*_1_*p*_0_)(*d*_5_−*d*_6_)+ *l*_2_−*l*_1_	*c*_4_−*c*_3_+*d*_0_*d*_5_*d*_8_*g*_2_(*d*_7_−1)+ (*d*_1_−*d*_2_)*h*_2_+*d*_5_*d*_8_*m*_2_*p*_0_(*d*_7_−1)	*a*_1_*b*_1_*n*_2_(*b*_5_−*b*_4_)+*c*_6_−*c*_5_+*d*_0_*d*_5_*d*_7_*g*_1_(1−*d*_8_) +*d*_0_*d*_5_*d*_7_*g*_2_(1−*d*_8_)+*d*_0_*d*_5_*d*_7_(*d*_8_−1) +*d*_5_*d*_7_*m*_3_*p*_0_(*d*_8_−1) $+f_{2}\left[\sin\left(\frac{\Pi d_{3}}{2}\right)-\sin\left(\frac{\Pi d_{4}}{2}\right)\right]+$ *n*_0_*u*_0_(*u*_2_−*u*_1_)	(*, *, *)
*E*_8_(1, 1, 1)	*a*_1_*b*_5_*n*_1_(*b*_2_−*b*_1_)+*c*_1_−*c*_2_+ (*d*_0_*d*_7_*d*_8_*g*_1_+*d*_7_*d*_8_*m*_1_*p*_0_)(*d*_6_−*d*_5_)+ *l*_1_−*l*_2_	*c*_4_−*c*_3_+*d*_0_*d*_6_*d*_8_*g*_2_(*d*_7_−1)+ (*d*_1_−*d*_2_)*h*_2_+*d*_6_*d*_8_*m*_2_*p*_0_(*d*_7_−1)	*a*_1_*b*_2_*n*_2_(*b*_5_−*b*_4_)+*c*_6_−*c*_5_+*d*_0_*d*_6_*d*_7_*g*_1_(1−*d*_8_) +*d*_0_*d*_6_*d*_7_*g*_2_(1−*d*_8_)+*d*_0_*d*_6_*d*_7_(*d*_8_−1) +*d*_6_*d*_7_*m*_3_*p*_0_(*d*_8_−1) $+f_{2}\left[\sin\left(\frac{\Pi d_{3}}{2}\right)-\sin\left(\frac{\Pi d_{4}}{2}\right)\right]$ +*n*_0_*u*_0_(*u*_2_−*u*_1_)	(*, *, *)

The equilibrium points (^*^,^*^,^*^) in Table 4 represent that each eigenvalue (λ_1_, λ_2_, λ_3_, ) may be >0, may be equal to 0, or may be < 0. In an evolutionary game, a stabilization point occurs only if all eigenvalues are < 0. Revenue components of medical institutions: revenue from implementing strategies + subsidies – compensation payments – penalties – losses due to unreasonable fees; revenue components of platforms: operational revenue + rewards for detecting safety hazards – compensation payments – penalties – costs of implementing strategies; revenue components of insurance companies: revenue from claims efficiency – costs of implementing strategies – losses due to unreasonable fees – compensation payments – penalties – losses due to fraudulent insurance policies Losses due to insurance fraud. By observing and analyzing Table 4, we can obtain the stable measurement combinations of the evolutionary game under the model we constructed for the eight scenarios (see Strategies 1–8).

Strategy 1 When the three eigenvalues corresponding to *E*_1_ in Table 3 are all < 0 (λ_1_ < 0, λ_2_ < 0, λ_3_ < 0), there exists a stabilization point in the replicated dynamic equation *E*_1_(0, 0, 0). λ_1_ < 0 in *E*_1_ represents that medical institutions have higher benefits under a fully anonymous information sharing strategy than those obtained under a partially anonymous information sharing strategy; λ_2_ < 0 represents that platforms have higher benefits under a loosely regulated strategy than those obtained under a tightly regulated strategy; and λ_3_ < 0 represents that insurance companies have higher benefits under a no-audit strategy than those obtained under an audit strategy. At this time, the strategies of each subject are: fully anonymous information sharing by healthcare providers, loose regulation by platforms, and no auditing by insurance companies.

Strategy 2 When the three eigenvalues corresponding to *E*_2_ in Table 3 are all < 0 (λ_1_ < 0, λ_2_ < 0, λ_3_ < 0), there exists a stabilization point in the replicated dynamic equation *E*_2_(0, 0, 1). λ_1_ < 0 in *E*_2_ represents that medical institutions have higher benefits under a fully anonymous information sharing strategy than those obtained under a partially anonymous information sharing strategy; λ_2_ < 0 represents that platforms have higher benefits under a loosely regulated strategy than those obtained under a tightly regulated strategy; and λ_3_ < 0 represents that insurance companies have lower benefits under a no-audit strategy than those obtained under an audit strategy. At this time, the strategies of each subject are: fully anonymous information sharing by healthcare providers, loose regulation by platforms, and auditing by insurance companies.

Strategy 3 When the three eigenvalues corresponding to *E*_3_ in Table 3 are all < 0 (λ_1_ < 0, λ_2_ < 0, λ_3_ < 0), there exists a stabilization point in the replicated dynamic equation *E*_3_(0, 1, 0). λ_1_ < 0 in *E*_3_ represents that medical institutions have higher benefits under a fully anonymous information sharing strategy than those obtained under a partially anonymous information sharing strategy; λ_2_ < 0 represents that platforms have lower benefits under a loosely regulated strategy than those obtained under a tightly regulated strategy; and λ_3_ < 0 represents that insurance companies have higher benefits under a no-audit strategy than those obtained under an audit strategy. At this time, the strategies of each subject are: fully anonymous information sharing by healthcare providers, strict regulation by platforms, and no auditing by insurance companies.

Strategy 4 When the three eigenvalues corresponding to *E*_4_ in Table 3 are all < 0 (λ_1_ < 0, λ_2_ < 0, λ_3_ < 0), there exists a stabilization point in the replicated dynamic equation *E*_4_(1, 0, 0). λ_1_ < 0 in *E*_4_ represents that medical institutions have lower benefits under a fully anonymous information sharing strategy than those obtained under a partially anonymous information sharing strategy; λ_2_ < 0 represents that platforms have higher benefits under a loosely regulated strategy than those obtained under a tightly regulated strategy; and λ_3_ < 0 represents that insurance companies have higher benefits under a no-audit strategy than those obtained under an audit strategy. At this time, the strategies of each subject are: partially anonymous information sharing by healthcare providers, loose regulation by platforms, and no auditing by insurance companies.

Strategy 5 When the three eigenvalues corresponding to *E*_5_ in Table 3 are all < 0 (λ_1_ < 0, λ_2_ < 0, λ_3_ < 0), there exists a stabilization point in the replicated dynamic equation *E*_5_(1, 1, 0). λ_1_ < 0 in *E*_5_ represents that medical institutions have lower benefits under a fully anonymous information sharing strategy than those obtained under a partially anonymous information sharing strategy; λ_2_ < 0 represents that platforms have lower benefits under a loosely regulated strategy than those obtained under a tightly regulated strategy; and λ_3_ < 0 represents that insurance companies have higher benefits under a no-audit strategy than those obtained under an audit strategy. At this time, the strategies of each subject are: partially anonymous information sharing by healthcare providers, strict regulation by platforms, and no auditing by insurance companies.

Strategy 6 When the three eigenvalues corresponding to *E*_6_ in Table 3 are all < 0 (λ_1_ < 0, λ_2_ < 0, λ_3_ < 0), there exists a stabilization point in the replicated dynamic equation *E*_6_(1, 0, 1). λ_1_ < 0 in *E*_6_ represents that medical institutions have lower benefits under a fully anonymous information sharing strategy than those obtained under a partially anonymous information sharing strategy; λ_2_ < 0 represents that platforms have higher benefits under a loosely regulated strategy than those obtained under a tightly regulated strategy; and λ_3_ < 0 represents that insurance companies have lower benefits under a no-audit strategy than those obtained under an audit strategy. At this time, the strategies of each subject are: partially anonymous information sharing by healthcare providers, loose regulation by platforms, and auditing by insurance companies.

Strategy 7 When the three eigenvalues corresponding to *E*_7_ in Table 3 are all < 0 (λ_1_ < 0, λ_2_ < 0, λ_3_ < 0), there exists a stabilization point in the replicated dynamic equation *E*_7_(0, 1, 1). λ_1_ < 0 in *E*_7_ represents that medical institutions have higher benefits under a fully anonymous information sharing strategy than those obtained under a partially anonymous information sharing strategy; λ_2_ < 0 represents that platforms have lower benefits under a loosely regulated strategy than those obtained under a tightly regulated strategy; and λ_3_ < 0 represents that insurance companies have lower benefits under a no-audit strategy than those obtained under an audit strategy. At this time, the strategies of each subject are: fully anonymous information sharing by healthcare providers, strict regulation by platforms, and auditing by insurance companies.

Strategy 8 When the three eigenvalues corresponding to *E*_8_ in Table 3 are all < 0 (λ_1_ < 0, λ_2_ < 0, λ_3_ < 0), there exists a stabilization point in the replicated dynamic equation *E*_8_(1, 1, 1). λ_1_ < 0 in *E*_8_ represents that medical institutions have lower benefits under a fully anonymous information sharing strategy than those obtained under a partially anonymous information sharing strategy; λ_2_ < 0 represents that platforms have lower benefits under a loosely regulated strategy than those obtained under a tightly regulated strategy; and λ_3_ < 0 represents that insurance companies have lower benefits under a no-audit strategy than those obtained under an audit strategy. At this time, the strategies of each subject are: partially anonymous information sharing by healthcare providers, strict regulation by platforms, and auditing by insurance companies.

## 5 Evolutionary game simulation analysis

This study employs MATLAB to simulate evolutionary game data, aiming to elucidate the influence of medical institutions, big data innovation platforms, and insurance companies on game stability. Parameter values are derived from existing literature and specifically tailored using insights from the “Research Report on the Collaborative Innovation Model of Commercial Health Insurance and Pharmaceuticals” alongside the “Securities Research Report on Medicare Individual Accounts Purchasing Commercial Insurance Functions Online and the Anticipated Acceleration of Health Insurance Development,” among others. Setting parameters *a*_1_ = 0.5, *b*_1_ = 0.6, *b*_2_ = 0.5, *b*_4_ = 0.6, *b*_5_ = 0.4, *c*_1_ = 10, *c*_2_ = 15, *c*_3_ = 15, *c*_4_ = 20, *c*_5_ = 15, *c*_6_ = 20, *d*_0_ = 100, *d*_1_ = 0.3, *d*_2_ = 0.6, *d*_3_ = 0.6, *d*_4_ = 0.8, *d*_5_ = 0.4, *d*_6_ = 0.5, *d*_7_ = 0.5, *d*_8_ = 0.5, *f*_2_ = 20, *g*_1_ = 1/3, *g*_2_ = 1/3, *h*_2_ = 15, *l*_1_ = 10, *l*_2_ = 5, *m*_1_ = 0, *m*_2_ = 0, *m*_3_ = 0, *p*_0_ = 50, *u*_0_ = 0.2, *u*_1_ = 0.5, *u*_2_ = 0.3, *n*_0_ = 80*n*_1_ = 20, *n*_2_ = 20, accordingly. Utilizing the specified parameter settings, we examine the stability of the eight strategies outlined in the preceding section, focusing on the roles of medical institutions, platforms, and insurance companies within the evolutionary game. A critical inquiry is whether mechanisms such as platform monitoring, insurance company auditing, and governmental reward-and-punishment policies can enhance the propensity of medical institutions to share information when their initial willingness is minimal. Additionally, it is pertinent to investigate other factors that influence the functionality of the information chain and the decision-making proclivity of each entity. This section analyzes these dimensions through various parameters.

### 5.1 Stabilization points under different strategies

The system exhibits multiple evolutionary paths. To explore the evolutionary process of each stakeholder and to verify the correctness and validity of the model based on evolutionary stability results, three groups comprising *E*_1_, *E*_2_, *E*_3_, *E*_4_, *E*_5_, *E*_6_, *E*_7_, and *E*_8_ are selected for numerical simulation using eight equilibrium points of pure strategy. Initial values (randomized between 0 and 1) are provided. To ensure the rationality of the original parameter settings, the model parameters must satisfy economic assumptions and empirical determinations. Based on the practical significance of the model parameters and previous research experience, the parameter settings are as shown above.

#### 5.1.1 Stable points under strategies 1–4

**Figure 2:**
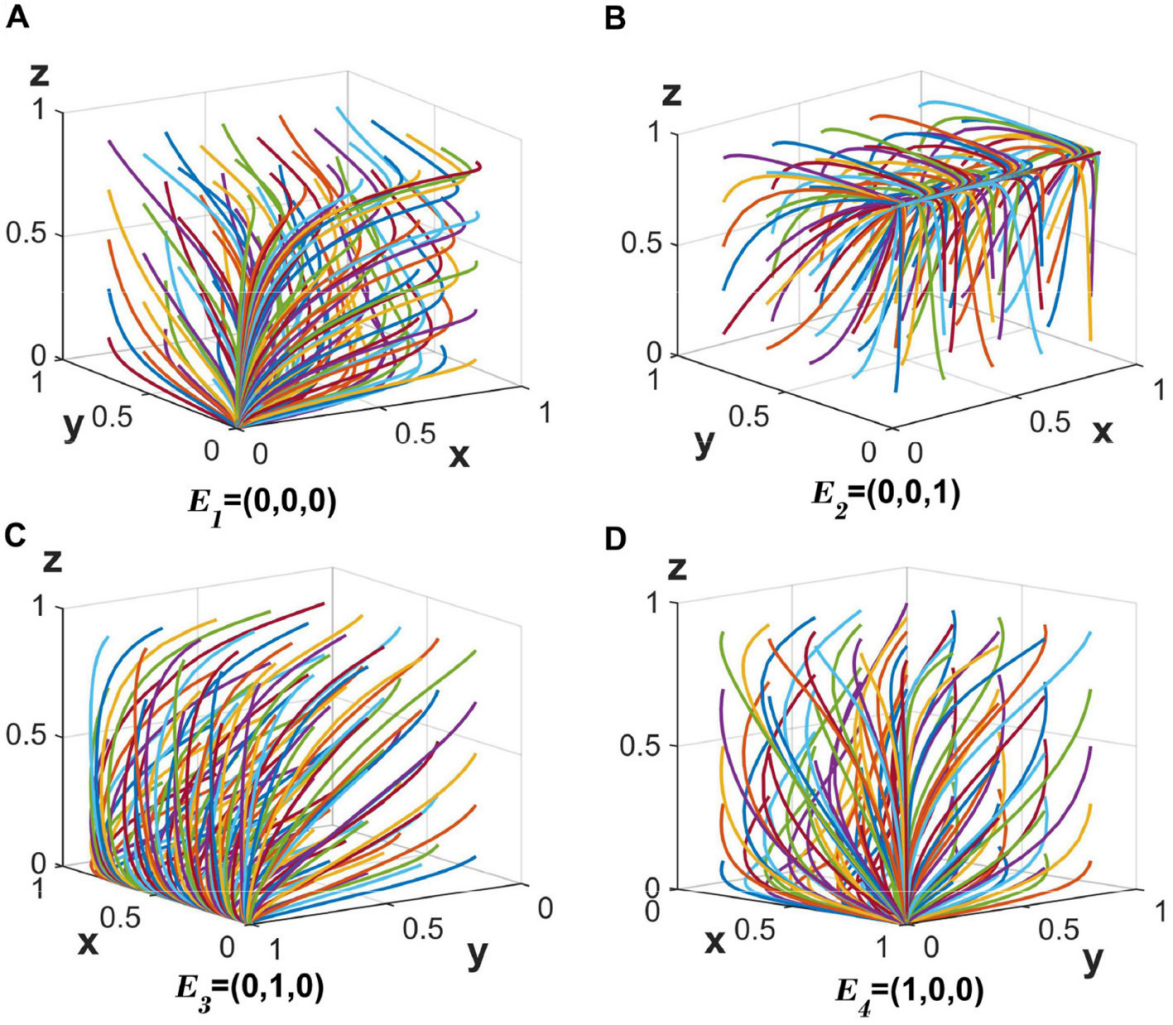
**(A–D)** The evolution of the tripartite participants toward a stable equilibrium of strategies 1–4.

When the values are set to *c*_4_ = 30, *c*_6_ = 30 , and the other parameters remain unchanged, the corresponding eigenvalues for *E*_1_ are all negative (λ_1_ < 0, λ_2_ < 0, λ_3_ < 0), indicating stability at point *E*_1_(0, 0, 0). The stabilizing strategy for the three parties is {fully anonymous information sharing, loose regulation, no auditing}, with the evolutionary trajectory depicted in Figure 2A.

When the value is set to *c*_4_ = 30 while others stay constant, the eigenvalues associated with *E*_2_ become negative (λ_1_ < 0, λ_2_ < 0, λ_3_ < 0), leading to stability at *E*_2_(0, 0, 1). The resulting strategy is {fully anonymous information sharing, loose regulation, auditing}, as shown in Figure 2B.

Changing only *c*_6_ = 30 maintains the other parameters, resulting in negative eigenvalues for *E*_3_ (λ_1_ < 0, λ_2_ < 0, λ_3_ < 0), and stabilization at *E*_3_(0, 1, 0). The stable strategy here is {fully anonymous information sharing, strict regulation, no auditing}, illustrated in Figure 2C.

Finally, setting _*c*_2_ = 20, *c*4_ = 30, *c*_6_ = 30, *d*_0_ = 50 with other parameters unaltered yields negative eigenvalues for *E*_4_ (λ_1_ < 0, λ_2_ < 0, λ_3_ < 0). This leads to stabilization at *E*_4_(1, 0, 0), characterized by the strategy {partially anonymous information sharing, loose regulation, no auditing}, as displayed in Figure 2D.

#### 5.1.2 Stable points under strategies 5–8

**Figure 3:**
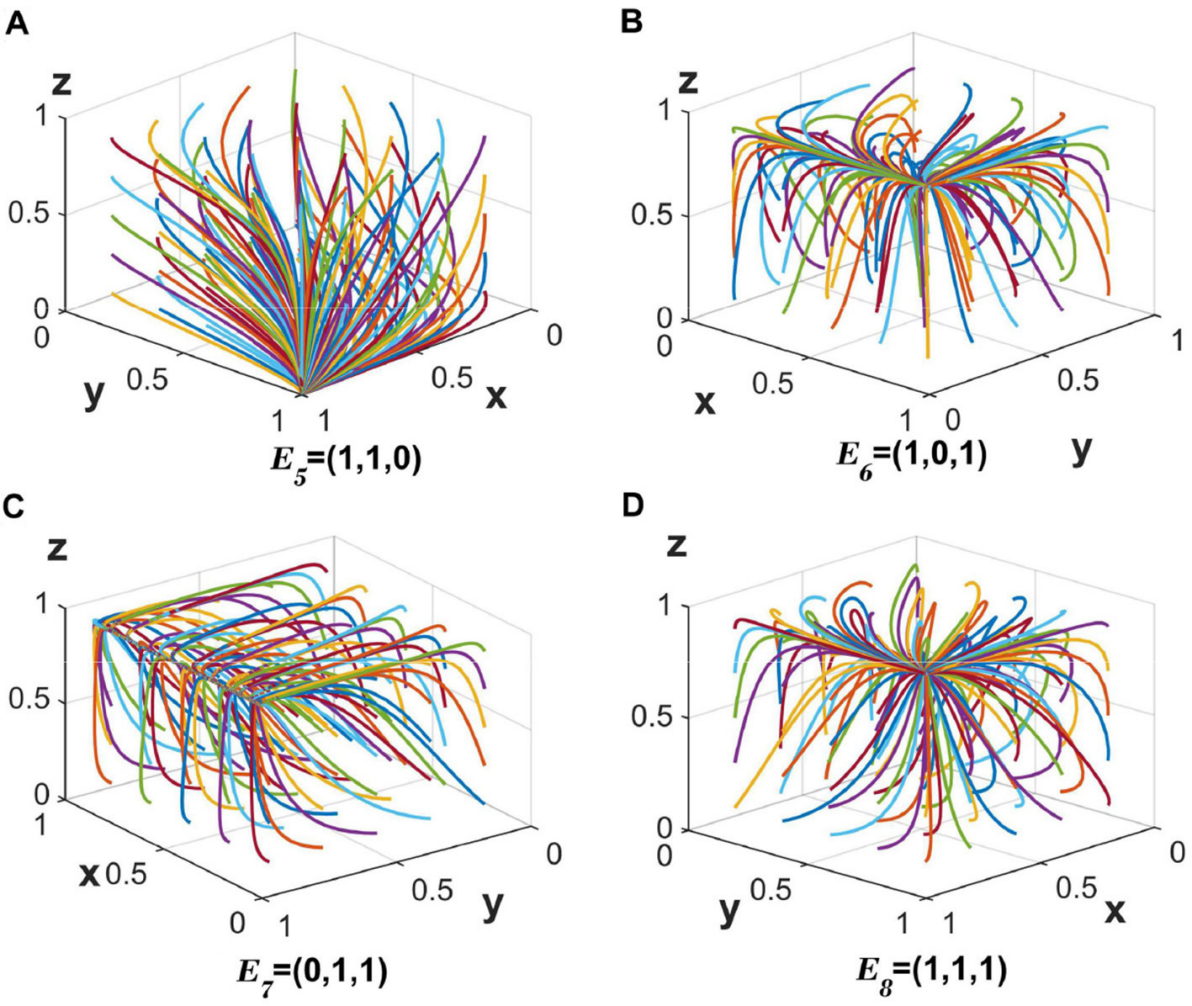
**(A–D)** The evolution of the tripartite participants toward a stable equilibrium of strategies 5–8.

When the value of _*c*_2_ = 20, *c*6_ = 30 is changed and the other values remain unchanged, the corresponding three eigenvalues corresponding to *E*_5_ are all < 0 (λ_1_ < 0, λ_2_ < 0, λ_3_ < 0), and strategy 5 reaches the stabilization point *E*_5_(1, 1, 0). The stabilizing evolutionary strategy of the three parties is {partially anonymous information sharing, strict regulation, no auditing}, and the evolutionary trajectory is shown in Figure 3A.

When the values are changed to _*c*_2_ = 20, *c*4_ = 30 and the other values remain unchanged, the corresponding three eigenvalues corresponding to *E*_6_ are all < 0 (λ_1_ < 0, λ_2_ < 0, λ_3_ < 0), and strategy 6 reaches the stabilization point *E*_6_(1, 0, 1). The stabilizing evolutionary strategy of the three parties is {partially anonymous information sharing, loose regulation, auditing}, and the evolutionary trajectory is shown in Figure 3B.

When all parameters take constant values, the three eigenvalues corresponding to *E*_7_ are all < 0 (λ_1_ < 0, λ_2_ < 0, λ_3_ < 0), and the strategy 7 reaches the stabilization point *E*_7_(0, 1, 1). The stabilizing evolutionary strategy of the three parties is {fully anonymous information sharing, strict regulation, auditing}, and the evolutionary trajectory is shown in Figure 3C.

When the values are changed to *c*_2_ = 20, *l*_2_ = 7 and the other values remain unchanged, the corresponding three eigenvalues corresponding to *E*_8_ are all < 0 (λ_1_ < 0, λ_2_ < 0, λ_3_ < 0), and strategy 8 reaches the stabilization point *E*_8_(1, 1, 1). The stable evolution strategy of the three parties is {partially anonymous information sharing, strict regulation, auditing}, and the evolution trajectory is shown in Figure 3D.

### 5.2 Impact of changes in government penalization policies and the severity of penalties on different subjects

#### 5.2.1 The impact of government punitive policies on data breaches caused by medical institutions themselves

**Figure 4:**
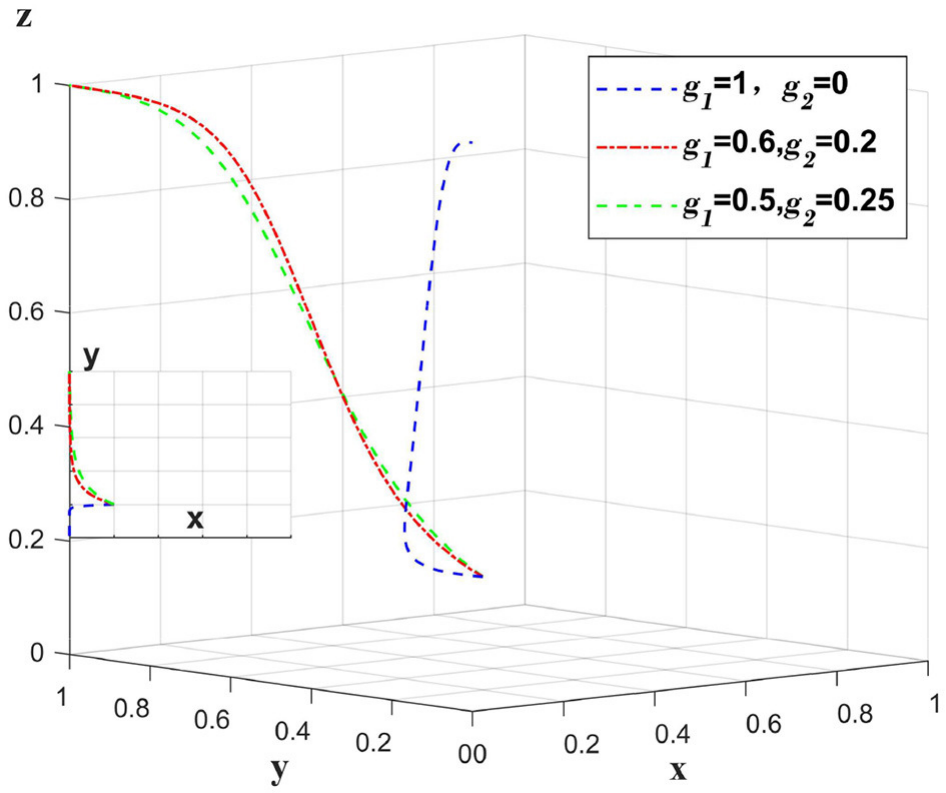
Impact of medical institutions taking varying responsibility for data breaches caused by themselves.

When a data breach occurs due to a medical institution, the medical institution bears the main compensation amount, and *g*_1_ represents the proportion of the compensation amount borne by the medical institution. The government punishes the medical institution, at this time set *m*_1_ = 1. Only change the value of *g*_1_, *g*_2_, *m*_1_, other values remain unchanged, analyze the change of the main body in the case of the whole system bears the different proportion of the compensation amount. As shown in Figure 4.

When a data breach occurs due to a medical institution, if the compensation amount is fully paid by the medical institution, the platform will choose loose regulation due to a “fluke mind.” By sharing a certain amount of compensation between the platform and the insurance company, the platform can shift to strict regulation. However, excessive sharing of the compensation amount will reduce the incentive of insurance companies to implement auditing strategies. Figure 4 illustrates that when a data breach occurs due to a medical institution, having the platform and insurance company bear a certain percentage of the compensation plays a supervisory role, and oversharing will reduce the motivation of the platform and insurance company.

#### 5.2.2 The impact of government punitive policies on data breaches caused by platforms themselves

**Figure 5:**
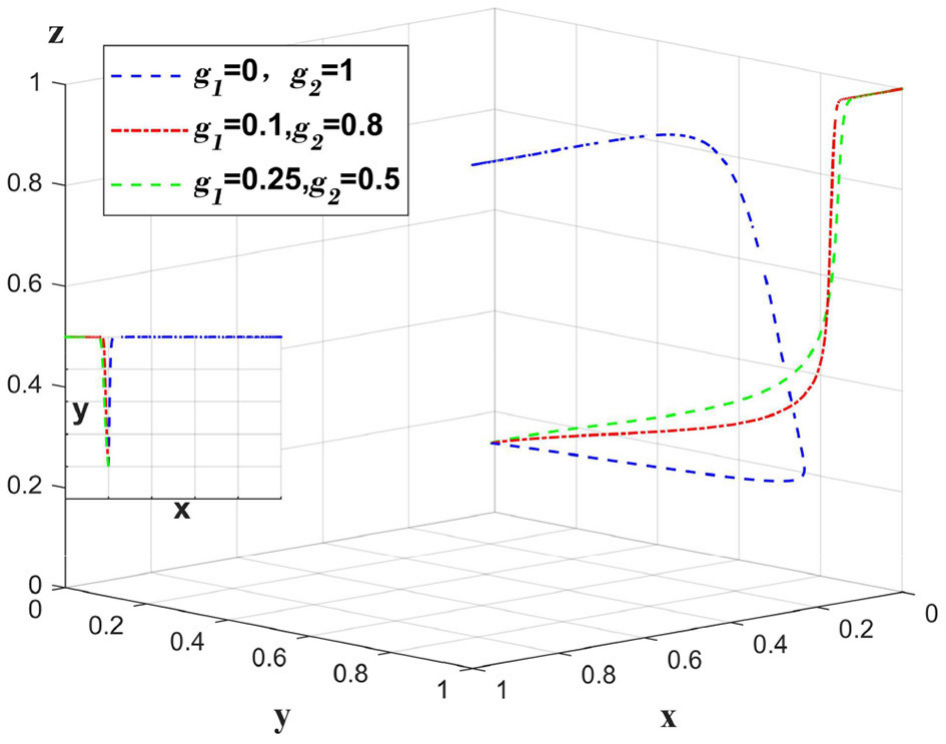
Impact of platforms taking varying responsibility for data breaches caused by themselves.

When there is a data breach due to the platform, the platform bears the main compensation amount, and *g*_2_ represents the proportion of the compensation amount borne by the platform. The government punishes the platform, at this time set *m*_2_ = 1. Only change the value of *g*_1_, *g*_2_, *m*_2_, other values remain unchanged, and analyze the change of the main body when the whole system bears the different proportion of the compensation amount. As shown in Figure 5.

When a data breach occurs due to a platform, if the compensation amount is fully paid by the platform, the medical institution will choose a partially anonymous information sharing strategy in order to gain more benefits. Medical institutions and insurance companies bear a certain amount of compensation, and the liability sharing system deters medical institutions from continuing to choose a partially anonymous information sharing strategy, causing them to shift to a fully anonymous information sharing strategy. Excessive sharing of the compensation amount does not increase the motivation of insurance companies to implement the auditing strategy and medical institutions to implement the fully anonymous information sharing strategy.

#### 5.2.3 The impact of government punitive policies on data breaches caused by insurance companies themselves

**Figure 6:**
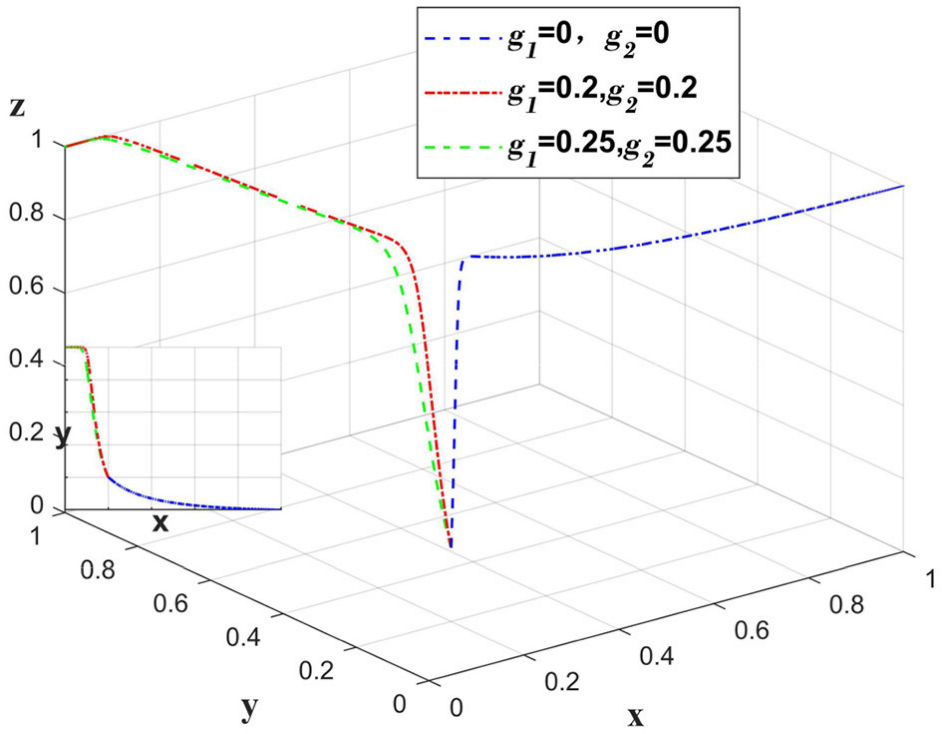
Impact of insurance companies taking varying responsibility for data breaches caused by themselves.

When the data breach occurs due to the insurance company, the insurance company bears the main compensation amount, 1−*g*_1_−*g*_2_ represents the proportion of the compensation amount borne by the insurance company. The government punishes the insurance company, at this time, set *m*_3_ = 1. Only change the values of _*g*_1_, *g*_2_, *m*3_, other values remain unchanged, and analyze the change of each subject when the whole system bears the different proportion of the compensation amount. As shown in Figure 6.

When a data breach occurs at an insurance company, if the compensation amount is fully covered by the insurance company, medical institutions will choose a partially anonymous information-sharing strategy to maximize their benefits. If the medical institution and the platform share a certain amount of compensation, the liability sharing system will discourage the medical institution from continuing to choose a partially anonymous information-sharing strategy and make the medical institution change to a fully anonymous information sharing strategy. Excessive sharing of compensation will not increase the motivation of platforms to implement auditing strategies and medical institutions to implement fully anonymous information sharing strategies.

#### 5.2.4 Data breaches caused by external factors

**Figure 7:**
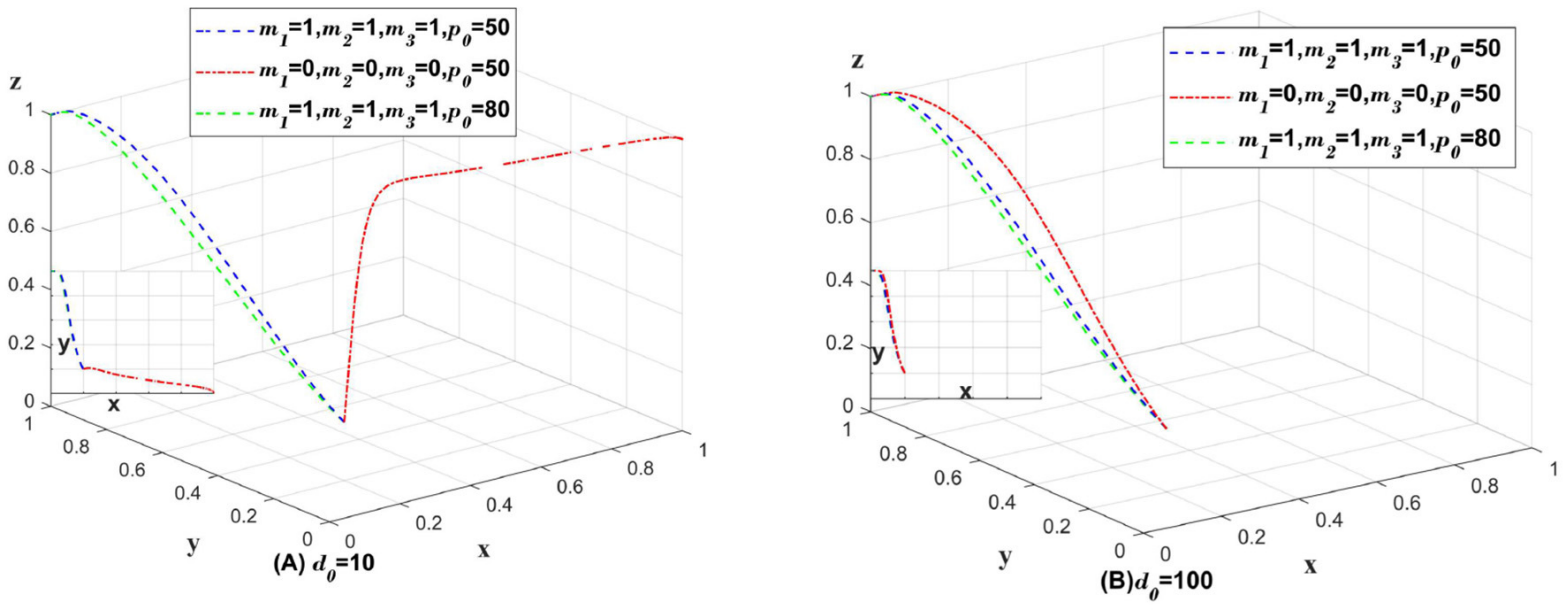
Impact of changes in penalization policies on data breaches caused by externalities [**(A)** for small data breaches; **(B)** for large data breaches].

Explore how the government's punishment policy should be implemented when the data breach is caused by external factors and it is not known which subject caused the data breach. When *m*_1_, *m*_2_, *m*_3_ is 1, it means that the government should penalize medical institutions, platforms and insurance companies even though the cause of data breach cannot be found. When *m*_1_, *m*_2_, *m*_3_ is 1, it means that the government does not penalize the three subjects; *p*_0_ represents the amount of penalty for the three subjects; and *d*_0_ represents the compensation to consumers. Only change the values of *m*_1_, *m*_2_, *m*_3_, *p*_0_, *d*_0_, the rest of the parameter values remain unchanged. As shown in Figure 7. In Figure 7A, *d*_0_ = 10, which represents the case of data breach in a small range. In Figure 7B, *d*_0_ = 100, which represents the case of data breach in a large range.

As illustrated in Figure 7A, when a minor data breach occurs, the lack of penalties for medical institutions, platforms, and insurance companies can lead to these entities opting for a partially anonymous information-sharing strategy to maximize profits. The profits gained from this strategy are then used to cover the compensation costs. In such scenarios, governmental penalties against these three entities can incentivize medical institutions to adopt a fully anonymous information-sharing strategy, thereby promoting the development of the entire system. However, if all three entities are penalized, increasing the fine amount may reduce the incentive for insurance companies to implement auditing strategies.

As illustrated in Figure 7B, large-scale data breaches necessitate substantial compensation from medical institutions, platforms, and insurance companies. Consequently, these entities recognize the severe repercussions of data breaches, which diminishes the efficacy of governmental punitive measures. Large-scale data breaches infringe upon legal boundaries and are not merely moral issues or matters that can be resolved through government fines alone. They require accountability in accordance with local laws. In this context, heightened fines and stringent government penalties not only fail to bolster systemic oversight but also dampen the incentives for these organizations to adopt proactive data protection strategies.

### 5.3 Impact of changes in government subsidies on different subjects

**Figure 8:**
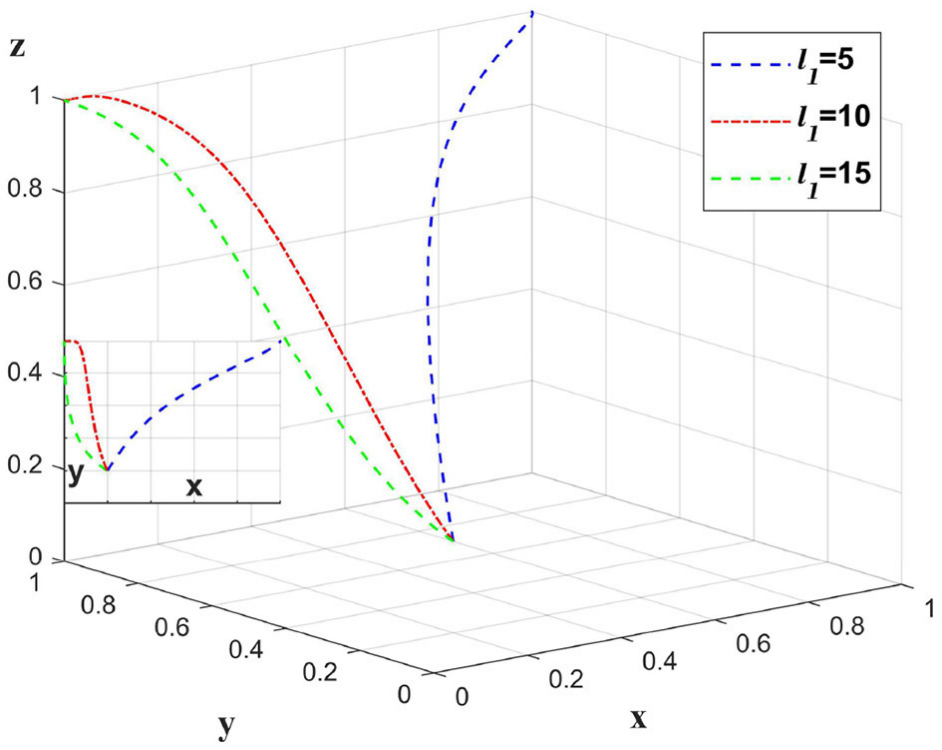
The impact of changes in subsidies to medical institutions on decision-making.

Medical institutions are worried about the data breach problem caused by medical data information sharing, and the subsidy policy in this paper only subsidizes medical institutions. Only the value of *l*_1_ is changed, and other values remain unchanged to analyze the impact on the whole chain when medical institutions are subsidized. As shown in Figure 8.

To optimize clarity, concision, and academic style, the paragraph has been revised as follows: When medical institutions receive a modest subsidy, they tend to abandon strategies with lower data breach probabilities in favor of higher profits, opting instead for partially anonymous information-sharing strategies. As subsidies increase, these institutions are more likely to adopt fully anonymous information sharing and select strategies with minimal chances of data breach detection. However, excessive government subsidies can diminish the motivation for platforms to implement stringent regulatory measures and for insurance companies to undertake thorough audits, thereby exerting a negative influence on overall governance.

### 5.4 Impact of changes in claims efficiency on different subjects

**Figure 9:**
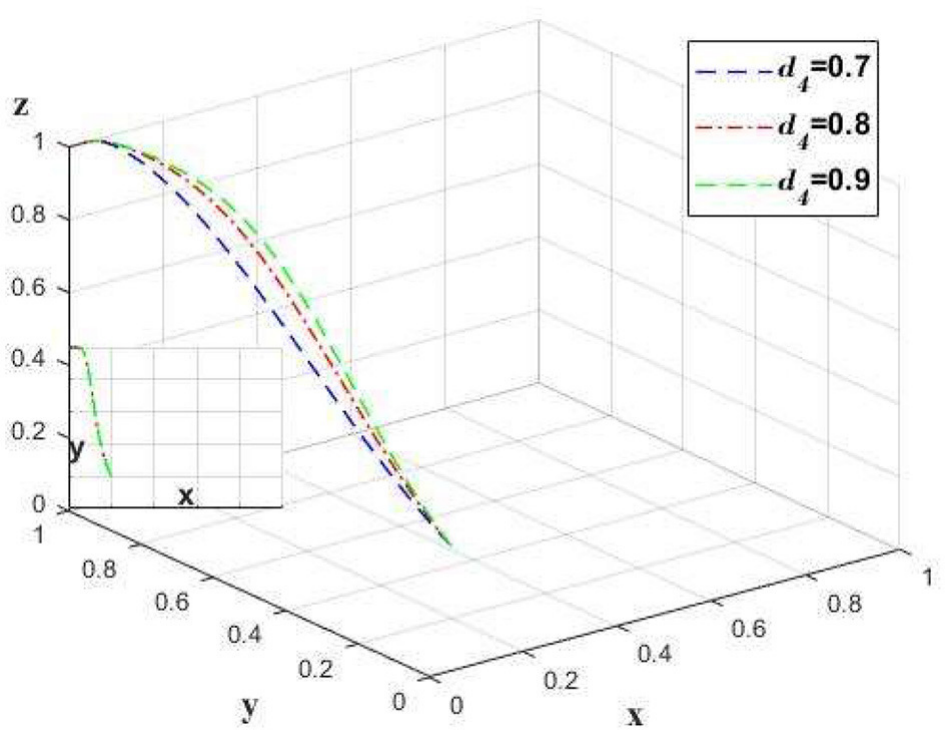
The impact of changes in claim settlement efficiency on strategies.

The implementation of auditing in insurance companies will increase the efficiency of claims processing. Change only the value of *d*_4_ and leave the other values unchanged to analyze the claims efficiency of the insurance company, and the impact on the chain as a whole. As shown in Figure 9.

The adoption of audit mechanisms by insurance companies can improve claim settlement efficiency. As illustrated in Figure 9, there is a positive correlation between claim efficiency and the propensity of insurance companies to undertake audits. However, this correlation diminishes at higher levels of efficiency. Once a certain level of efficiency is reached, additional enhancements do not significantly influence the inclination of medical institutions. At this point, insurance companies should not expend excessive resources to achieve optimal claim speed; rather, they could adopt a strategy where *d*_4_ = 0.8, which not only helps improve their own profitability but also ensures the willingness of the entire supply chain in finance to the greatest extent.

### 5.5 The impact of changes in the probability of insurance fraud on different entities

The implementation of audits by insurance companies reduces the probability of insurance fraud. Change only the value of *u*_2_ and leave the other values unchanged to analyze the probability of insurance company cheating on the insurance policy, and the impact on the whole chain. This is shown in Figure 9.

**Figure 10:**
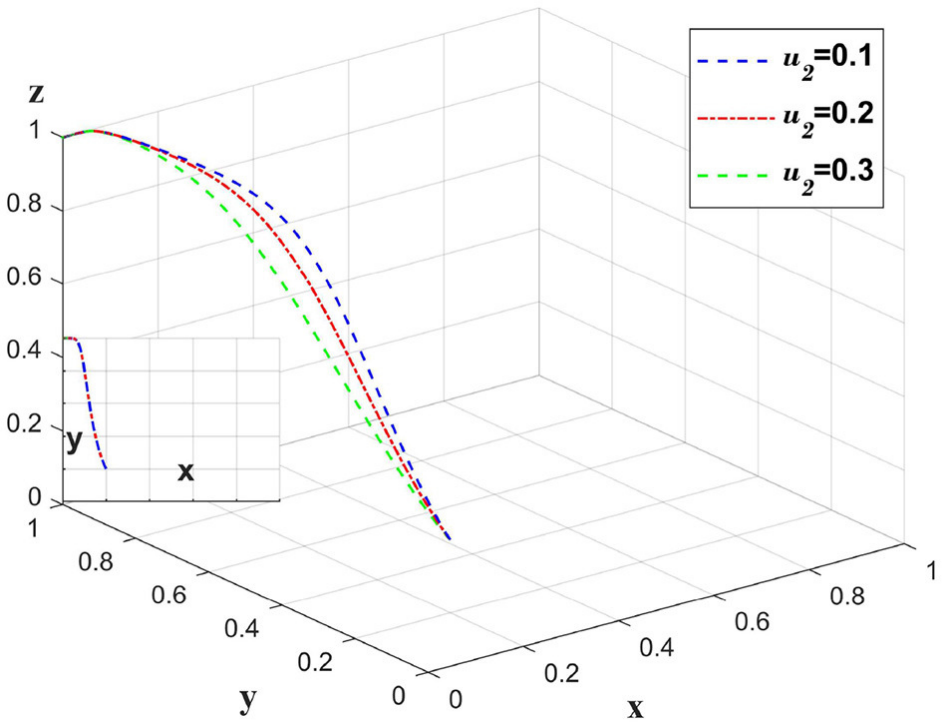
The impact of changes in the probability of insurance fraud on strategies.

As seen in Figure 10. Upon implementing an audit mechanism, insurance companies observe a significant reduction in the willingness to continue audits due to an increased probability of insurance fraud, thereby augmenting the platform's demand for stringent oversight. From the standpoint of medical institutions, extreme probabilities of fraud, whether high or low, diminish their propensity to actively share information. When insurance companies carry out audits, they can maintain the probability at *u*_2_ = 0.3, where the willingness of medical institutions to actively share information is at its highest, and the platform's willingness for strict oversight is also greater than at *u*_2_ = 0.2. In this scenario, insurance companies can save on cost inputs and achieve an optimal solution.

### 5.6 The impact of changes in the probability of unreasonable charges on different entities

**Figure 11:**
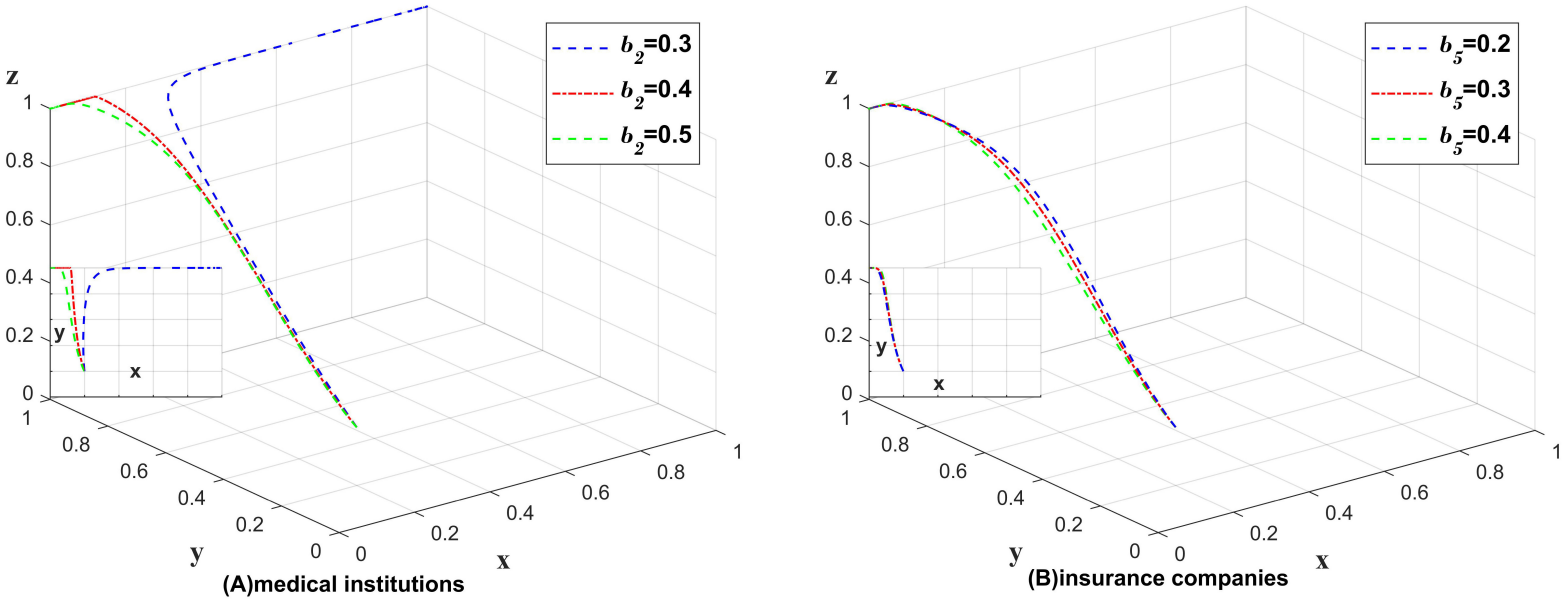
No impact of changes in the probability of unreasonable charges on strategies was found. **(A)** Medical institutions **(B)** insurance companies.

Unreasonable charges affect medical institutions and insurance companies, Figure 11A shows the impact of the change in the probability of undetected unreasonable charges for medical institutions on different subjects, and Figure 11B shows the impact of the change in the probability of undetected unreasonable charges for insurance companies on different subjects. The impact of unreasonable charges on the whole chain is analyzed by changing only the values of *b*_2_, *b*_5_ and leaving the other values unchanged. As shown in Figure 11.

Figure 11A shows the impact of the change in the probability of not detecting unreasonable charges in medical institutions on different subjects. If the probability of detecting unreasonable charging phenomenon can be substantially increased under the partially anonymous information sharing strategy, medical institutions will ignore the impact of data breach and choose the partially anonymous information sharing strategy. Figure 11B shows the impact of the change in the probability of not detecting unreasonable charges in medical institutions on different subjects. A significant increase in the probability of detecting unreasonable charges under the insurance company's choice of auditing strategy does not bring significant benefits and positive changes to the chain. Consequently, we conclude that medical institutions are primarily responsible for the detection of unreasonable charges, while insurance companies play a secondary role. Medical institutions can afford to invest more in detecting unreasonable charges, whereas insurance companies should not prioritize this issue excessively, as doing so would incur higher costs without achieving the desired benefits.

## 6 Discussion

This study employs evolutionary game theory to examine the promotion of mutual development between medical institutions and insurance companies through healthcare data sharing. The model is constructed based on factors such as data breaches, government rewards and penalties, claim processing efficiency, insurance fraud, and unreasonable fees. It proposes a cost-sharing mechanism for data breaches. We discuss the stability of the model and validate the theoretical framework through numerical simulations. Additionally, this paper explores the factors influencing the evolution of the healthcare data sharing system.

### 6.1 Conclusion

Our research has yielded several important conclusions. First, while most existing studies focus on the allocation of responsibility to individual entities (36), this study emphasizes a cost-sharing mechanism among multiple entities and proposes a more comprehensive framework for responsibility allocation. When breaches are triggered by medical institutions, they tend to seek co-sharing between platforms and insurance companies, which promotes platforms to strengthen regulation and incentivizes insurance companies to perform audits. If the responsibility for the breach lies with either the platform or the insurance company, the burden-sharing system will encourage medical institutions to choose a fully anonymous information sharing strategy; otherwise, medical institutions will opt for partially anonymous information sharing for the greater benefit.

Second, numerous studies advocate for enhancing the complexity and stringency of medical data sharing protocols to bolster security (37). Minor data breaches can prompt governmental penalties against healthcare institutions, platforms, and insurance companies, incentivizing these entities to adopt fully anonymous information-sharing methodologies, thereby influencing the overall system. While escalating fines may deter insurance firms from deploying audit strategies, a balanced approach is crucial in penalty policy formulation. Conversely, during extensive data breaches, the efficacy of government penalties diminishes due to the substantial compensation obligations faced by all parties involved, rendering increased fines counterproductive and potentially dissuading proactive strategy implementation.

Third, moderate subsidies can incentivize medical institutions to share information more actively. In contrast to previous studies (38), excessive subsidies may actually weaken their motivation. When receiving a small subsidy, medical institutions tend to forsake the strategy of low data breach probability in favor of partially anonymous information sharing to maximize profit. With limited resources, they may prioritize economic benefits over data security. As the subsidy amount increases, institutions are more likely to adopt fully anonymous information sharing and lower data breach probability. Sufficient financial incentives can encourage institutions to implement prudent data handling practices, thereby reducing the risk of data breaches. Although government subsidies aim to promote secure data management strategies, over-subsidization may backfire by diminishing the incentives for platforms to choose strictly regulated strategies and for insurance companies to conduct audits.

Fourth, the audit mechanisms of insurance companies enhance claim processing efficiency. However, as efficiency increases, the growth in their willingness to conduct audits gradually diminishes. There is an efficiency threshold beyond which further improvement does not significantly increase medical institutions' motivation to share information. Therefore, insurance companies should strive to find the optimal balance between cost and efficiency.

Fifth, an increase in insurance fraud probability diminishes the auditing enthusiasm of insurance companies while augmenting the regulatory zeal of platforms. An optimal level of insurance fraud probability exists that maximizes both the information sharing by medical institutions and the regulatory willingness of platforms. Consequently, insurance companies should maintain the probability of insurance fraud at this optimal level to optimize cost-effectiveness.

Sixth, the probability of detecting unreasonable charges significantly impacts both medical institutions and insurance companies. Compared to previous studies (39), our findings indicate that medical institutions tend to overlook the potential risk of data breaches, prioritizing the adoption of partially anonymous information-sharing strategies when such strategies substantially increase the likelihood of identifying unjustified billing. In their pursuit of economic efficiency and regulatory compliance, medical institutions may compromise a certain level of data privacy protection.

Although the implementation of audit strategies by insurance companies enhances the detection of unreasonable charges, it does not yield significant efficiency gains or motivate the various actors in the healthcare chain effectively. Medical institutions should be the primary force in detecting unreasonable charges and should allocate more resources to this area, while insurance companies should play a supportive role and avoid considering this issue as a central concern to prevent over-investment with limited returns. This means that tasks should be rationally allocated to ensure maximum efficiency with limited resources. For medical institutions, although there is a cost associated with detecting unreasonable charges, such an investment is justifiable given the potential benefits, including avoiding financial losses and maintaining reputation. Conversely, for insurance companies, prioritizing this issue may result in costs outweighing the benefits.

### 6.2 Theoretical findings

The objective of this study is to investigate the mechanisms for fostering collaboration between healthcare institutions and commercial health insurers through the sharing of medical data. Methodologically, the research employs evolutionary game theory, predicated on the assumption that both healthcare providers, digital platforms, and insurance entities exhibit bounded rationality. In constructing the model, variables such as irrational billing practices within medical settings, the efficacy of insurance claim processing, data security concerns among stakeholders, and the implementation of incentive and punitive measures in response to data breaches were incorporated. Results indicate that elements like inappropriate charges, claim processing efficiency, and the presence of rewards and penalties significantly impact the evolutionary trajectory of medical data-sharing behaviors. This investigation contributes not only methodological insights and modeling frameworks for enhancing cooperation between healthcare entities and commercial insurers but also broadens the application domains and theoretical boundaries of evolutionary game theory. Future research avenues might involve the utilization of more sophisticated mathematical models to precisely characterize collaborative interactions between healthcare institutions and commercial insurers, and exploring quadruple games or other advanced gaming approaches to delve deeper into the cooperative dynamics of these parties.

### 6.3 Management insights

Drawing from the analysis, several managerial implications emerge to foster medical institution and commercial health insurance development via healthcare data sharing. Firstly, this study introduces a novel multi-object cost-sharing mechanism in medical information security. By delineating responsibilities for data breaches and instituting a rational cost-sharing framework, it incentivizes active regulatory and auditing participation, facilitating fully anonymous information exchange in healthcare settings. This bolsters data protection, ensuring service quality and safety.

Secondly, policy penalties are essential for monitoring minor data breaches. However, in cases of major breaches, compensation should be prompt, tempered by avoiding overly stringent governmental sanctions that could deter long-term incentives for full anonymity in data sharing. Governments are advised to adopt a balanced, flexible approach in regulation, securing data while fostering information flow for a thriving healthcare sector.

Thirdly, moderate subsidies can stimulate medical institutions' engagement in information sharing, crucial for optimizing resource allocation. Careful subsidy amount determination is vital to avoid detrimental effects of excessive or insufficient funding, enhancing overall service efficiency, innovation, and technological advancement in medicine.

Fourthly, insurance companies must enhance claim processing efficiency through optimized audit processes, yet remain cautious against overinvestment in audit technologies to prevent diminishing returns. Striking this balance is key to operational efficacy across the healthcare ecosystem, alleviating institutional burdens, and strengthening patient trust in insurance services.

Fifthly, fraudulent claims impair insurers' audit enthusiasm and platform regulation. Managing insurance fraud probability optimally ensures audit effectiveness and minimizes unnecessary expenses, vital for a stable and healthy insurance market.

Lastly, intensifying audits on unreasonable charges aids insurers in cost control and encourages fair pricing and transparency within medical institutions. Addressing such charges seriously fosters a fairer, more transparent healthcare market, boosting patient confidence, insurance company credibility, and competitiveness, thereby advancing medical information sharing.

### 6.4 Potential ethical concerns

Due to disparities in size, service quality, and other aspects between smaller and larger medical institutions, stringent penalties may deter smaller entities from participating in information sharing. This paper introduces a collaborative liability framework wherein the government imposes penalties for minor data breaches. For significant breaches, the costs are shared among medical institutions, digital platforms, and insurance companies, alleviating the burden on smaller institutions without governmental penalties. Large-scale data breaches infringe upon legal boundaries and are not merely moral issues or matters that can be resolved through government fines alone. They require accountability in accordance with local laws. Furthermore, the Chinese government is spearheading an initiative alongside various organizations to establish a unified big data innovation platform, ensuring regional medical data conformity and facilitating cross-regional information sharing.

### 6.5 Limitations and future directions

Although evolutionary game theory has been employed to analyze how medical data sharing fosters collaboration between healthcare providers and commercial insurers, several limitations persist. The model, while reflecting real-world conditions closely, fails to encompass all intricacies and variances inherent in practice. Future empirical research can be conducted to test the theoretical predictions of the model and validate the effectiveness of its assumptions. Moreover, the investigation predominantly adopts a micro-level viewpoint, concentrating on the dynamics of cooperation promotion via data exchange, neglecting broader macroeconomic and political influences. Subsequent research should delve into these overarching factors' impact on data sharing and collaborative frameworks. Additionally, the study overlooks the burgeoning role of technologies like blockchain and AI in shaping data-sharing practices and their consequent effects on partnerships within the sector. Future inquiries are encouraged to incorporate these technological trends' influence on sharing behaviors and their transformative potential for collaboration paradigms.

## Data Availability

The original contributions presented in the study are included in the article/supplementary material, further inquiries can be directed to the corresponding author.
